# Expert predictions of changes in vegetation condition reveal perceived risks in biodiversity offsetting

**DOI:** 10.1371/journal.pone.0216703

**Published:** 2019-05-08

**Authors:** Josh Dorrough, Steve J. Sinclair, Ian Oliver

**Affiliations:** 1 Ecosystem Management Science Branch, Science Division, Office of Environment and Heritage New South Wales, Merimbula, New South Wales, Australia; 2 Arthur Rylah Institute for Environmental Research, Department of Environment, Land, Water and Planning, Heidelberg, Victoria, Australia; 3 Ecosystem Management Science Branch, Science Division, Office of Environment and Heritage New South Wales, Gosford, New South Wales, Australia; Bowling Green State University, UNITED STATES

## Abstract

Biodiversity offsetting typically involves the trade of certain losses of habitat with uncertain future conservation benefits. Predicting the latter requires estimates of two outcomes; the biodiversity losses without conservation management (averted loss), and the biodiversity gains with conservation management (management gain). However, because empirical data to inform these estimates are limited, they are normally guided by expert opinion, often derived via unstructured methods without consideration of uncertainty. Here we used a structured elicitation with 29 experts to gather estimates of averted loss and management gain at offset sites. We used two methods; (i) experts estimated change in an aggregate biodiversity value (vegetation condition) and; (ii) experts provided probabilistic estimates of change for individual vegetation condition attributes, such as the richness and cover of plant growth forms. On average, experts predicted there would be only modest improvements with conservation management, yet uncertainty and variation among experts was large; in some cases, conservation benefits were not predicted. Estimates of change in vegetation condition suggested that benefits were from both averted loss and management gains and were thought to most likely arise in cases where starting condition was low to moderate. Similar patterns were observed for individual vegetation condition attributes, with management gains, relative to a reference, tending to be negatively correlated with starting value. Our study finds that: (i) on average, gains at offset sites are expected to be small, (ii) at many sites, experts do not believe gains can be obtained, and (iii) experts’ opinions can be divergent resulting in elevated levels of uncertainty. The potential for losses under conservation management highlights the need to: identify those components of biodiversity most likely to benefit from conservation management; better understand those situations when offset obligations are most likely to be met and conversely those situations with higher risk; and further develop offset mechanisms that encourage early or prior gains. These findings together with the global proliferation of biodiversity offsetting, provide a strong imperative to improve empirical data and investment in long-term, site-based monitoring of biodiversity outcomes at offset sites.

## Introduction

Globally, there is increasing adoption of no net loss (NNL) policies and biodiversity offsetting for managing the impacts of human development on biodiversity [1, 2]. Under these schemes the stated objectives are usually to “offset” the residual negative impacts of development activities (those remaining after avoidance and minimisation of impact) by securing and managing habitat at another location with the intent to improve habitat quality or increasing the population size of target species [2]. Improvements, or “gains”, at those locations are expected to result from active management of threats and pressures (e.g. control of invasive plant species, better control of grazing by domestic and feral herbivores, reduction in water extraction for irrigation, cessation of fertiliser application) and from restoration (typically replanting or re-introduction of missing biodiversity elements). Notwithstanding broader concerns about biodiversity offsetting and the trading of nature [3], the contribution of offsets to the success of NNL objectives requires that gains are additional to any that might be expected in the absence of the offsets [1, 4], the gain is secured (i.e. on-going threat management and adequate protected status) [1] and the risks of not meeting the expected gains are minimised [1, 2].

A major challenge for NNL is that the biodiversity trades usually require exchange of known, certain losses with uncertain future gains [5]. To minimise risk, it has been argued that the offset gains should be achieved prior to the development impact [5, 6] but offset schemes almost always rely on gains being achieved after the development impact. This requires the ability to predict change, and real ecological consequences hinge on the accuracy of those predictions.

**Fig 1:**
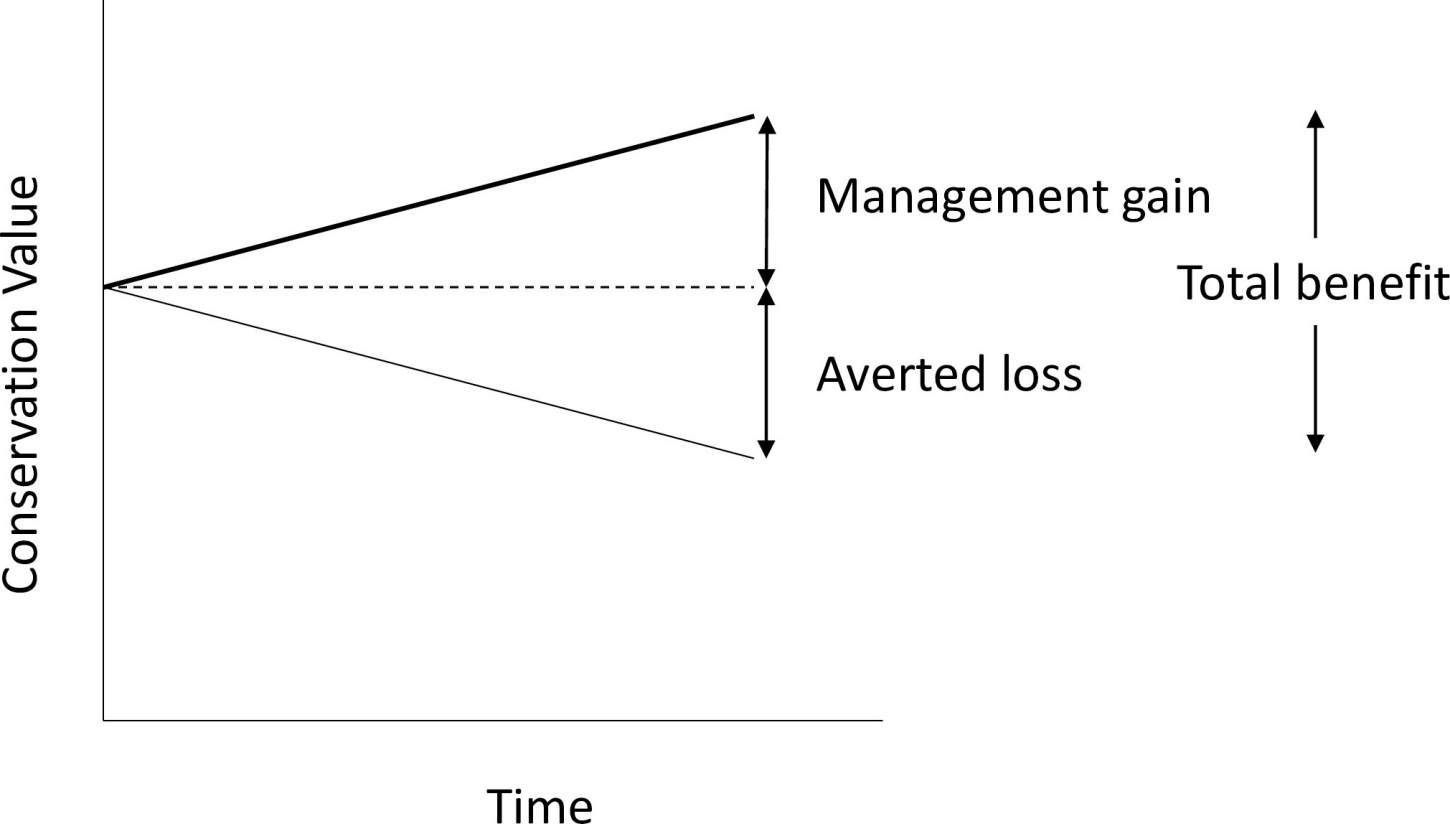
The total benefit of implementing conservation management is the sum of averted losses and additional gains above the current value. The top solid line represents trends in conservation value with offset and the lower solid line represents the trends under the counterfactual. While our elicitations focused on estimating management gain, where possible we also estimated averted loss and total benefit. Adapted from Maron et al. [7].

Maron and colleagues [7] proposed a simple model for estimating the total future benefit of conservation actions based on estimating the change in the biodiversity indicator with and without an offset (Fig 1). Total benefit is the sum of two separate components, management gain (MG) and averted loss (AL). Management gain is estimated as the future improvement relative to the current value at t_0_ (MG = future value with offset–current value). Averted loss is estimated in relation to a counterfactual, those changes in biodiversity that are predicted to have occurred if the site were not managed to deliver an offset [1], and is the absolute expected future losses that would occur in the absence of an offset (AL = current value–future value without offset). The choice and quantification of the counterfactual is fundamental to estimating the potential benefits arising from conservation actions at offset sites. In most cases the counterfactual is assumed to be an on-going background rate of decline in biodiversity [8].

Much attention has been paid to the principles underlying NNL [2, 9, 10], but relatively little to the actual quantification of changes under offset schemes [2]. This is surprising given the predicted magnitude of change is often used directly to determine the scope of the offset in the field (e.g. its size and the degree of investment in its management). Because the outcomes of conservation management and restoration are highly variable, hard to predict and difficult to generalise [11, 12], the risks of not achieving adequate compensatory gains, within an appropriate time-frame, can be considerable. Estimating plausible future values and the uncertainty around them is a requirement in some offset schemes [13–15], although in other circumstances offset schemes use fixed multipliers or “rules of thumb” rather than explicit estimates [16]. Importantly, because estimates are rarely derived in a structured and consistent manner they are open to manipulation and inconsistency [17]. While acknowledgment of this issue is widespread, few studies have attempted to quantify the ecological outcomes of conservation management at offset sites in part because of a lack of generalisable data, models or monitoring of sufficient duration [10].

Because we have little data to inform predictions of future changes in biodiversity value, expert elicitation becomes a useful tool for developing hypotheses for where and when offsets have greatest potential for generating benefits. Expert judgment is heavily relied upon in many areas of environmental decision making, especially when knowledge is incomplete, there is substantial uncertainty and urgent predictions are required, precluding reliance on long-term experiments or monitoring [18]. Most elements of offset design are dependent to some extent on belief, opinion and value judgement, and informal expert opinion guides many aspects of NNL policies (i.e. choice of metrics, equivalency and location rules, predictions of benefits from conservation actions, estimates of uncertainty and time lags). However, rarely are offset provisions informed by a structured and explicit elicitation of opinion and belief, which considers variation and uncertainty.

Here we report the results of an expert elicitation with 29 vegetation field ecologists, practitioners, research scientists and ecological consultants on their predictions about the likely changes in biodiversity indicators in response to management of threats and pressures at hypothetical biodiversity offset sites. While expert opinion will not substitute for empirical data, it can highlight important sources of variation and provide useful guidance for identifying when and where offsetting has most potential for success but also where we should be most wary, and where empirical data are most urgently needed. We expected large variation among experts in their absolute predictions, but we hypothesised that experts would share similar opinions about those attributes and scenarios where benefits from offsetting would most likely accrue.

## Methods

### Identification and selection of experts

Our aim was to select a pool of experts who had extensive practical and/or theoretical knowledge of vegetation dynamics in south-eastern Australia. From an initial pool of 94 native vegetation professionals, 39 experts were invited to self-rate their experience in vegetation survey and monitoring through an online questionnaire and asked to participate in one of three two-day workshops (see S1 File). In total, 37 experts completed the online survey, and 29 attended the workshops.

### The focal ecosystem

Our online survey revealed that most experts had experience in the ecology, management and monitoring of grassy woodlands (S1 File) and for this reason we chose to primarily focus the elicitation on this ecosystem. During the workshops opinions were also elicited for other vegetation types, although results did not reveal differences among them (S2 File).

The focal ecosystem for the elicitations was a “Western Slopes Grassy Woodland” (WSGW) [19] in the Brigalow Belt South bioregion [20], in northern NSW. Western Slopes Grassy Woodlands are listed under the Australian *Environment Protection and Biodiversity Conservation Act 1999* [21], and are a priority for conservation effort. The Brigalow Belt South bioregion also supports known mineral resources that are the subject of active and proposed extraction activities [22], and which may trigger offset requirements. The woodland occurs at moderate altitude (<700m) and rainfall (550-800mm per annum) on relatively fertile clay-loam soils [19]. It extends from north-eastern Victoria through to southern Queensland, typically along the inland (western) side of the Great Dividing Range [23]. In the Brigalow Belt South bioregion the overstorey is dominated by eucalypts (typically *Eucalyptus albens*, *E*. *melanophloia*) with a groundlayer of perennial C4 and C3 grasses and a diversity of forb species, the abundance and identity of which varies largely depending on prior landuse [24].

### Biodiversity variables and metrics

We assumed site-scale biodiversity was represented by a range of vegetation structure and composition indices common to contemporary biodiversity assessment methods (Table 1). Although these do not capture the full complexity of biodiversity or the values attached to nature [25, 26], the use of simple proxies based on vegetation metrics is an assumption common to many offset schemes [27]. Individual attributes are often standardised based on benchmark values estimated from actual or theoretical reference sites and then aggregated to generate an index of habitat quality or vegetation condition [28]. Despite reasonable concerns about the trading among attributes in aggregate scores of biodiversity value or vegetation condition [14], aggregate values are often the metric used to compare gains and losses within biodiversity offsetting. While concepts such as vegetation condition are inherently subjective [29] previous research has demonstrated that experts can generate consistent and reliable aggregate scores that represent biodiversity value and habitat quality [29, 30]. In this paper we consider both individual attributes and an expert generated aggregate index of vegetation condition.

**Table 1:** Individual vegetation condition attributes that formed the basis for the structured elicitation.

Foliage Cover (%)	Species Richness (/400m^2^)	Habitat Structure
Tree	Tree richness	Large trees^A^
Shrub	Shrub richness	Litter cover (%)
Grass & grass-like	Grass & grass-like richness	Length of logs (m)^B^
Forb	Forb richness	
Fern	Fern richness	

Only native species contributed to estimates of growth form cover or species richness. The foliage cover and species richness of each growth form were assumed to be estimated within a 20m x 20m plot while habitat attributes were measured in a 20m x 50 m plot.

^A.^The number of trees with a diameter at breast height >0.5m.

^B.^The lineal length (m) of woody debris with a diameter >0.1m.

### The elicitation

We used two separate approaches to elicit expert opinions about current values and future values in vegetation condition. Firstly, we captured expert point predictions about the current and future aggregate condition of a vegetation patch. This approach has been shown to usefully capture complex belief models of existing habitat value [29, 30], though the method has not yet been extended to future predictions. This elicitation exercise was completed by all 29 experts. Secondly, we elicited from twenty-five experts, the probability densities of future values of individual vegetation attributes. To do this we used the trial roulette or histogram method [31], which allowed experts to graphically generate a probability histogram to represent their belief about future values. We used this approach to extract the maximum information about central tendency, the range of predicted values, and the degree of spread.

#### Evaluating current and future aggregate vegetation condition

Our approach followed that of Sinclair and colleagues [29, 30] in that experts were asked to rank and score synthetic WSGW vegetation patches (‘sites’), based on their current and future contributions to persistence of biodiversity, assuming this could be captured by a single aggregate value representing vegetation condition. Unlike Sinclair et al. [29, 30] our primary aim was not to generate a metric for predicting vegetation condition but rather to explore expert opinion about change in the aggregate value over time. We sought to examine whether experts generated consistent current and future estimates for comparable sites and identify those covariates correlated with their predicted changes. A pool of 64 synthetic WSGW sites, each assumed to be 2ha, was informed by available full-floristic plot data for this bioregional vegetation type in the NSW BioNet database (www.bionet.nsw.gov.au). Each site description included an estimate of native vegetation cover within a 1.5km radius of the patch, the percent cover of invasive alien plant species [32], total alien plant ground cover, the dominant plant species in each of five growth forms (trees, shrub, forbs, grasses, ferns) and the current values of each of the 13 vegetation attributes (Table 1, see example in S3 File). Experts were asked to assume that these data had been estimated from a randomly placed 20m x 20m plot (richness and foliage cover of each growth form) or 20m x 50 m plot (habitat structure attributes). The 64 sites covered a wide range of conditions including those representing species-rich remnants through to disturbed patches dominated by exotic vegetation.

Experts were each provided with a randomly-drawn subset of 10 sites and five that were common to all experts. The five common sites were selected *a priori* by the authors and were assumed to represent a range from expected high through to expected low quality. Experts were not aware that their set included five common sites. These five sites were included to ensure that all experts were considering sites across the full spectrum of variation in the ecosystem, as a means of calibrating their judgements [29, 30].

The experts were initially asked to rank the sites in order of current value to persistence of biodiversity, not considering costs of management or potential for future changes (improvement or degradation) in the vegetation. Experts were then asked to provide each site with a vegetation condition score out of 100 where 100 represented highest value and 0 held no value to biodiversity. Experts were told that multiple sites could be given the same score, that scores could be distributed in any way they desired and that values of 0 and 100 did not have to be used.

Following estimation of current value, experts were asked to provide estimates of the vegetation condition score (out of 100) 20 years into the future. Experts were asked to estimate a future vegetation condition score for each site assuming they were managed under an offset agreement. An offset agreement was assumed to require that the site was permanently secured for conservation management and that all major site-based threats and pressures were managed, if practicable, to maximise biodiversity outcomes. This was assumed to include monitoring and management of weeds and exotic pests, management or exclusion of livestock grazing as appropriate to best achieve conservation outcomes, implementation of appropriate fire regimes and retention of all fallen timber and dead trees. Experts were asked to assume no seeding or supplementary planting were undertaken. This combination of management activities is typical of current mandatory offset obligations under the NSW *Biodiversity Conservation Act 2016* [33]. Experts at both the 2^nd^ and 3^rd^ workshops (16 experts) were also asked to provide a future condition score which reflected their best estimate if the sites in question were managed under a business as usual (BAU) scenario. BAU assumed a continuation of existing management practices.

#### Trial roulette elicitation of individual vegetation attribute probability distributions

The trial roulette method was used to obtain an expert’s distribution of predicted future outcomes for individual vegetation attributes under a BAU scenario and with a biodiversity offset. The trial roulette method is a form of fixed interval elicitation where experts specify upper (θ_max_) and lower (θ_min_) bounds and their best estimate [31] and the experts upper and lower interval (θ_min_,θ_max_) is divided into *n* equal sized bins. The expert is asked to distribute *n “*chips” (in this case *n* = 100) among the bins with the proportion of chips in a specific bin assumed to represent the probability of that outcome occurring.

We asked experts to specify lower (**θ**_min_) and upper (**θ**_max_) values for a given vegetation attribute such that the range **θ**_min_,**θ**_max_ represented an interval within which the experts were confident that the true value occurred. The interval was then automatically divided into 10 equal sized “bins”. Experts were asked to allocate the 100 “chips” into the 10 bins in a way that best represented their expectation of the likely distribution of values. To make this more tangible, experts were asked to imagine 100 “trials” of the specific scenario and estimate how many trials would end up in each of the 10 bins. The elicitation was undertaken in a spreadsheet with a linked histogram to enable experts to visually observe their allocations (S3 File). Previous work has shown that using graphical interfaces can substantially improve the accuracy of subjective distributions [34]. To familiarise experts with the trial histogram method, provide them with confidence in the task and a base level of calibration, each expert initially undertook five practice exercises derived from published studies (S3 File).

Experts were initially asked to generate probability distributions, for each vegetation attribute in Table 1 that reflected their opinion about the likely range of values they would expect in WSGW that were of reference condition. A reference condition in this context was conceived as the best-attainable condition in the contemporary landscape [35]. Experts were asked to assume that each of the 13 vegetation attributes were sampled in austral spring in a year of median rainfall within 20m x 20m plots (richness and foliage cover of each growth form) or 20m x 50m plots (habitat attributes), randomly placed within 2ha patches of relatively homogenous vegetation.

After completing their reference distributions, experts were asked to make predictions 20 years into the future for each of the 13 vegetation attributes, based on one or more scenarios. The scenarios for which future values were elicited are summarised in Table 2. For each scenario experts were provided with brief descriptions of a hypothetical 2ha site, including starting values of each of the vegetation attributes, monthly rainfall (median, 10^th^ and 90^th^ percentiles), estimates of the cover of invasive alien plant species, and dominant species in each growth form (see example in S3 File). Experts were asked to assume that the site occurred within a landscape with >70% native vegetation cover (in a 1.5km buffer around each patch) but was not within the current nature conservation estate. Each site was assumed to represent a relatively homogenous area of the same vegetation type and similar condition state.

**Table 2:** The three Western Slope Grassy Woodland (WSGW) scenarios for which future values of 13 vegetation attributes were elicited (also see S3 File).

Scenario	Vegetation Class	Bioregion	Initial Condition	Vegetation Condition	Gain component
1	Western Slopes Grassy Woodland	Brigalow Belt South	Moderate	60	AL, MG, TB
2	Western Slopes Grassy Woodland	Brigalow Belt South	Good	84	MG
3	Western Slopes Grassy Woodland	Brigalow Belt South	Poor	39	MG

For the WSGW in moderate condition future values were estimated for both the BAU and biodiversity offset scenarios, enabling calculation of averted loss (AL), management gain (MG) and total benefit (TB). Initial Condition is a qualitative and subjective description of the overall site quality, based on the opinion of the authors. Vegetation Condition was estimated from an ensemble boosted regression tree model of expert evaluations of current value of synthetic WSGW’s (see S7 File), where high values indicate greater value to biodiversity.

For one WSGW scenario, experts were asked to predict changes in the vegetation attributes after 20 years of BAU management, and assuming the patch was instead secured and managed as a biodiversity offset. This enabled calculation of averted loss, management gain and total benefit as per Fig 1. For all remaining scenarios, experts were only asked to make predictions assuming that patches were secured as biodiversity offsets, and so only management gain was calculated. This approach was taken to reduce expert fatigue during the workshops. We compared estimates of management gain among each of the three WSGW scenarios to see whether starting conditions influenced expert predictions.

### Data analysis

#### Aggregate vegetation condition

Management gain was estimated for each of the expert’s 15 vegetation patches. For those where BAU outcomes were estimated then averted loss and total benefit were also calculated. The distribution of expert evaluations for each of the 64 vegetation patches were inspected using box and whisker plots, sorting individual sites by their median current vegetation condition.

We used boosted regression trees (BRT) with the gbm.step function in the packages gbm [36] and dismo [37] within R [38] to undertake exploratory analysis of current vegetation condition, management gain, averted loss and total benefit. Covariates included all 13 vegetation attributes, landscape vegetation cover, invasive alien plant cover, total alien plant cover and expert identity. For models of management gain, averted loss and total benefit we included each expert's estimate of current vegetation condition as an additional covariate. Seventy five percent of the data were randomly selected for training each of the BRT models (bag fraction = 0.75) with the remaining data used for cross validation. Models were fit with a tree complexity of 5 and a learning rate of 0.005. Tree complexity, also known as interaction depth, sets the maximum number splits in each tree, while learning rate specifies the weight of each regression tree in the final model, with lower values ensuring that larger numbers of trees contribute to the final model. Boosted regression trees identify the relative importance of covariates by ranking each covariate based on the number of times it is selected for splitting, weighted by the squared improvement to the model of each split, and averaged over all trees. The relative influence of each covariate is then normalized to sum to 100, with higher numbers indicating a greater relative influence in describing variation in the response variable.

#### Distribution of individual vegetation attributes

Numeric distributions were fit to each of the expert’s binned data using the package SHELF [39] in R [38]. Fitting of numeric distributions enabled us to combine predictions when different θ_min_,θ_max_ were provided by different experts (and hence non-matching bins). Prior to fitting distributions, the elicited histograms were inspected for multi-modality. A range of parametric distributions were initially fit to the data (normal, log-normal, beta), however final parameter estimates were derived using either a beta distribution, for vegetation cover attributes estimated as a percentage and that could be re-scaled to the range (0,1), or a normal distribution for all remaining attributes.

One challenge when comparing different individual expert predictions is in understanding their frame of reference. The same absolute change in the value of a vegetation attribute may represent a perceived small or a large relative change for two different experts. To account for this difference we scaled the current and future attribute values using the experts elicited reference value:

$${BV}_{a}=\frac{V_{a}}{R_{a}}$$

where *BC*_*a*_ is the biodiversity score or “condition”, *V*_*a*_ is the estimate of the current or future value and *R*_*a*_ is the reference value for attribute *a*. This approach is comparable to the use of reference states or benchmarks for assessing relative biodiversity loss or restoration success [40–42] and is often employed in offset schemes to standardise loss and benefit [14]. We limited *BC*_*a*_ to a maximum value of 1 when *V*_*a*_ ≥ *R*_*a*_, which is a common assumption in assessing the relative quality of habitat or biodiversity [14]. While an attribute could continue to accrue above the reference value this may or may not represent an actual “gain” in biodiversity value. For each biodiversity attribute the averted loss, management gain and total benefit of the proposed offset can then be estimated relative to the reference on a scale of 0–1.

For each vegetation attribute, in a specific scenario, we combined estimates of *BC* across all experts. We did this by firstly taking 80–85 random draws from each expert's elicited probability distributions for the reference state, future with offset and future under business as usual (if estimated for that starting scenario). For each attribute we then estimated *BC* for the starting state (*BC*_*start*_), business as usual (*BC*_*bau*_) and future with offset (*BC*_*offset*_) and also calculated averted loss (*AL* = *BC*_*start*_—*BC*_*bau*_), management gain (*MG* = *BC*_*offset*_-*BC*_*start*_) and total benefit (*TB* = *AL* + *MG*) for each of the randomly drawn samples. Individual expert random samples of AL, MG and TB were then pooled, resulting in approximately 2100 randomly sampled estimates for each vegetation condition attribute that considered variation within and among each expert. Using these data, we explored, for a moderate condition WSGW, the distribution of AL, MG and TB for each vegetation attribute, except fern richness and cover. Ferns were excluded because they were often absent or of very low cover in expert reference distributions. We graphically explored variation in AL and MG for each attribute among individual experts and examined whether individual experts tended to be generally optimistic or pessimistic about future attribute trends, or whether their opinions differed depending on the attribute and management scenario (BAU or managed as an offset). We finally examined the distribution of MG in relation to *BC*_*star*t_ for each attribute, except fern cover and richness, among WSGWs with differing attribute starting values (Table 2, S3 File). These starting values approximated the authors interpretations of a WSGW in a remnant, lightly or ungrazed, unfertilized state; a WSGW in a moderately grazed state; and a heavily grazed and fertilized WSGW.

## Results

### An aggregate measure of biodiversity value, vegetation condition

#### Initial vegetation condition

**Fig 2:**
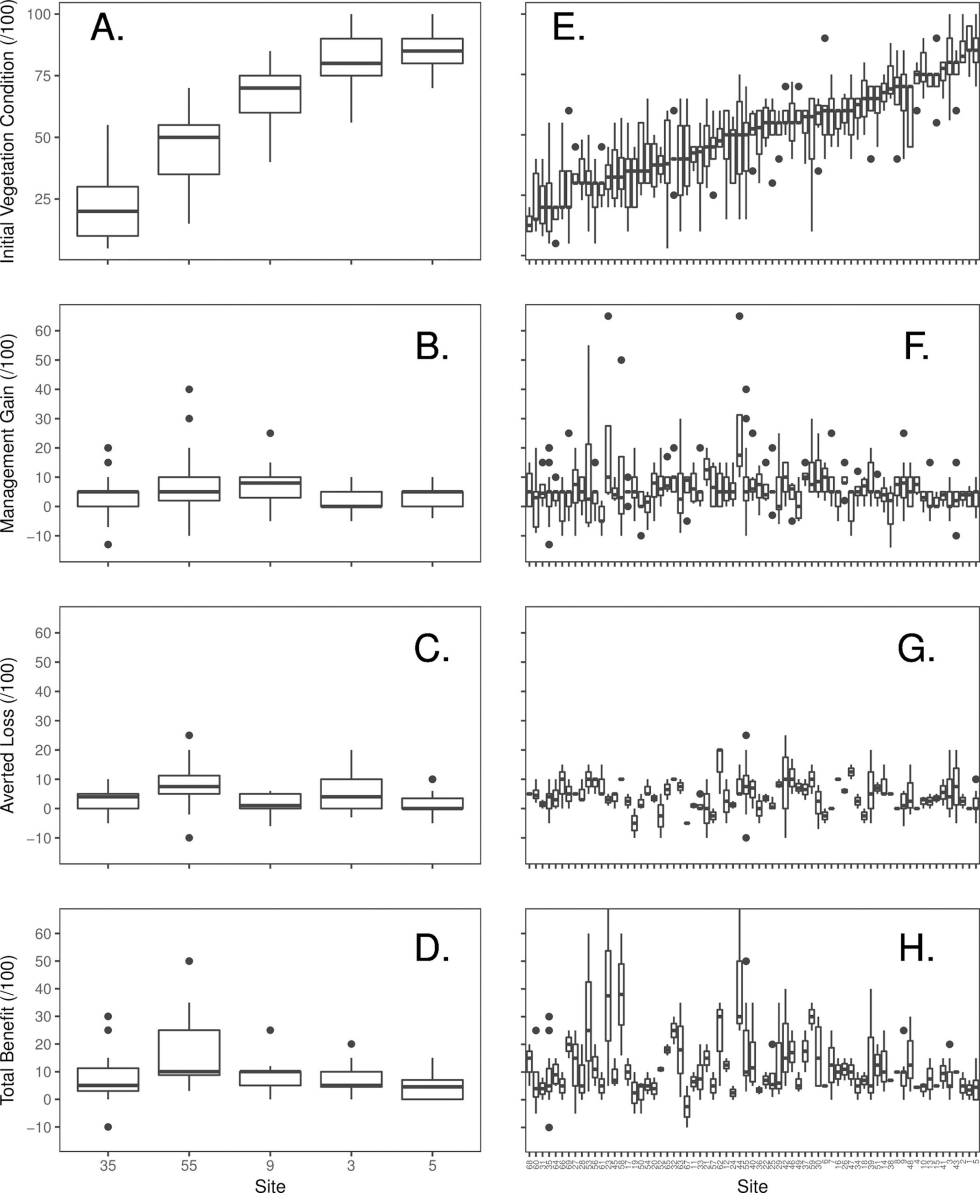
**Boxplots of expert estimates of vegetation condition (A, E), management gain (B, F), averted loss (C, G) and total benefit (D, H).** A through D are the five common vegetation patches provided to all 29 experts. Each patch in E through H was assessed by 4–6 experts. In all graphs the sites are ranked according to panel E, i.e. by their median current expert vegetation condition score. Averted loss and total benefit are based on estimates from 16 experts.

While there was variation in expert opinion of the aggregate value to biodiversity of each vegetation patch, rankings and aggregate scores were relatively consistent among the 29 experts (Fig 2A and 2E). A boosted regression tree of initial vegetation condition revealed that expert identity had the largest relative influence (a normalised relative influence, NRI, of 31) such that some experts tended to score all sites higher or lower than the average expert. Shrub cover (NRI = 22), grass cover (NRI = 20) and tree cover (NRI = 10) were also found to be relatively important in explaining expert judgements of vegetation condition. Expert estimates of vegetation condition tended to be positively correlated with all native vegetation attributes but negatively correlated with invasive alien plant cover (S4 File).

#### Future predictions of biodiversity value

Most experts predicted that MG would be relatively small (Fig 2B & 2F). Median expert estimates of MG across the 64 patches was 5 / 100, with lower 25^th^ and upper 75^th^ quartiles of 0 and 10 respectively. However, a single expert had median predictions of 25 / 100 and a maximum of 70 /100 (S5 File) while negative estimates of MG were also elicited from some experts (Fig 2B & 2F). The boosted regression tree models indicated that expert identity had the largest relative influence on management gain (NRI = 72), with only small contributions from initial vegetation condition (NRI = 15.4), invasive alien plant cover (NRI = 2.9) and landscape vegetation cover (NRI = 2.5). The data (Fig 2B & 2F) and BRT fitted functions (S4 File) suggest a negative, yet non-linear correlation between current biodiversity value and MG, a weak positive effect of landscape vegetation cover and a negative effect of invasive alien plant cover.

Averted loss (AL), derived from expert estimates of the change in vegetation condition under a BAU scenario, was estimated by 16 experts (Fig 2C & 2G). The median estimate of AL was 5, with upper and lower quartiles of 0 and 9 respectively under a BAU scenario. A single expert predicted that vegetation condition would weakly increase with BAU. Experts again were consistently optimistic or pessimistic in their estimate of averted loss (NRI = 60.3). Initial vegetation condition (NRI = 13.0), invasive alien plant cover (NRI = 13.2) and landscape vegetation cover (NRI = 4.2) each had only small to moderate relative influence in the BRT. Data and fitted functions from the BRT indicate a weak positive non-linear relationship between current biodiversity value and expert estimates of AL. Averted loss was greatest at moderate to high initial vegetation condition (Fig 2C & 2G, S4 File). Averted loss was also weakly negatively correlated with landscape vegetation cover and positively with HT weed cover.

Total benefit (TB) was estimated from values elicited from 16 experts. Median TB was 7.5 with lower (25^th^) and upper (75^th^) quartiles of 5 and 15 respectively. There was large among-expert variation in TB, evident in the normalised relative influence from the BRT (NRI = 66.3). Moderate relative influence was also associated with initial vegetation condition (NRI = 21.9) and a slight influence of invasive alien plant cover (NRI = 4.9). Data (Fig 2D & 2H) and the fitted functions from the BRT suggested that TB tended to be highest when initial vegetation condition was low to moderate (S4 File). There was little support for any correlation with either invasive alien plant cover or landscape vegetation cover (S4 File). The lack of effect of invasive alien plant cover or landscape vegetation cover was presumably because the magnitudes of AL and MG were similar and each was associated with opposing effects (ie. MG = +ve landscape vegetation cover, -ve invasive alien plant cover; AL = -ve landscape vegetation cover, +ve invasive alien plant cover).

### Individual attribute predictions from the trial roulette method

#### Averted loss, management gain and total benefit for a WSGW

**Fig 3:**
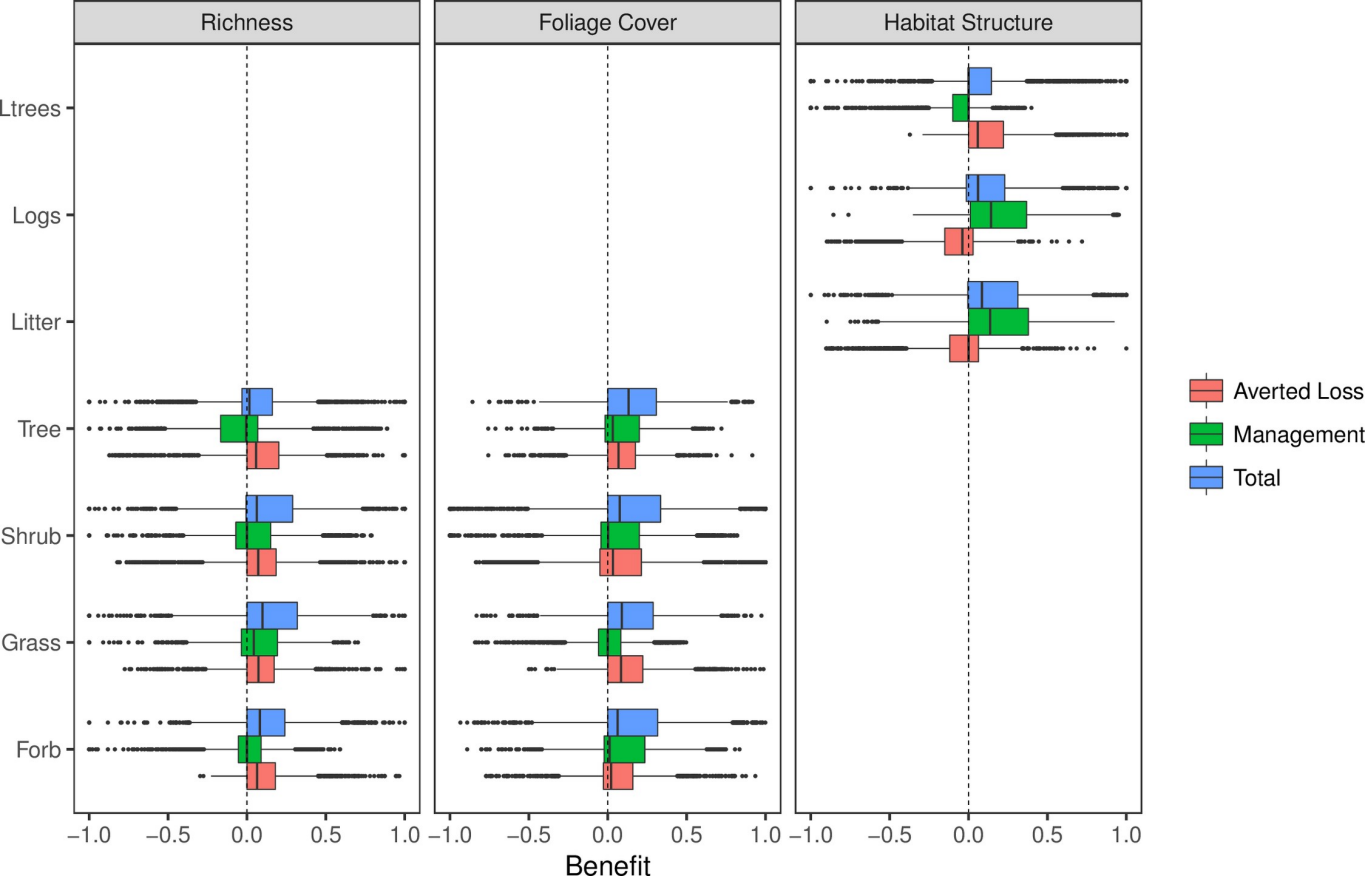
Boxplots of averted loss, management gain and total benefit after 20 years for individual vegetation attributes within a Western Slopes Grassy Woodland with moderate starting values. For each attribute, data are derived by pooling 2100 random draws from the future and reference probability distributions of 25 experts. Total benefit is the sum of averted loss and management gain. Averted loss is estimated as the difference between the counterfactual and the initial attribute condition and therefore takes a positive value when the attribute is expected to decline relative to the reference under a business as usual scenario. Ltrees = Large Trees.

For each vegetation condition attribute, we estimated the distribution of AL, MG and TB by randomly sampling from each expert’s reference, future under BAU and future with offset probability distributions. These random samples were pooled (2100 random samples for each attribute) and the resultant distributions of AL, MG and TB reflect both variation within and among experts (Fig 3, S6 File).

The lower quartile (25^th^ percentile) of AL estimates were >0 for the richness of all growth forms, the foliage cover of grasses and trees and the number of large trees (Fig 3). This suggests a tendency towards a consensus that these vegetation condition attributes will decline under a BAU scenario. Predictions of AL were less consistent for forb, shrub, length of fallen logs and litter cover (Fig 3).

Positive estimates of MG indicate an expert opinion that with an offset the attribute is expected to improve within 20 years, relative to the starting value. Both litter cover and the length of fallen logs were generally expected to increase following adoption of an offset (lower quartile is >0) while declines, or at best no change, in large trees numbers was thought to be most likely (Fig 3). Although some other attributes tended to have a median MG > 0 (e.g. tree and forb foliage cover and grass richness), predictions were uncertain owing to both inter- and intra-variation among experts (S6 File) and both declines and increases were predicted. In all cases, median predictions of absolute changes with management were never more than one species or 5% foliage cover.

The distribution of TB indicated that the sum of MG and AL were predicted to be mostly positive, except for large trees and tree richness (Fig 3). In most cases TB was dominated by AL. However, the results also indicated that TB can also often be negative, indicative of an expectation that even with an offset the attribute might decline relative to the counterfactual. Across all experts and all attributes, 25% of all randomly drawn estimates of TB were negative and a further 15% were 0, indicating no expected benefit.

**Fig 4:**
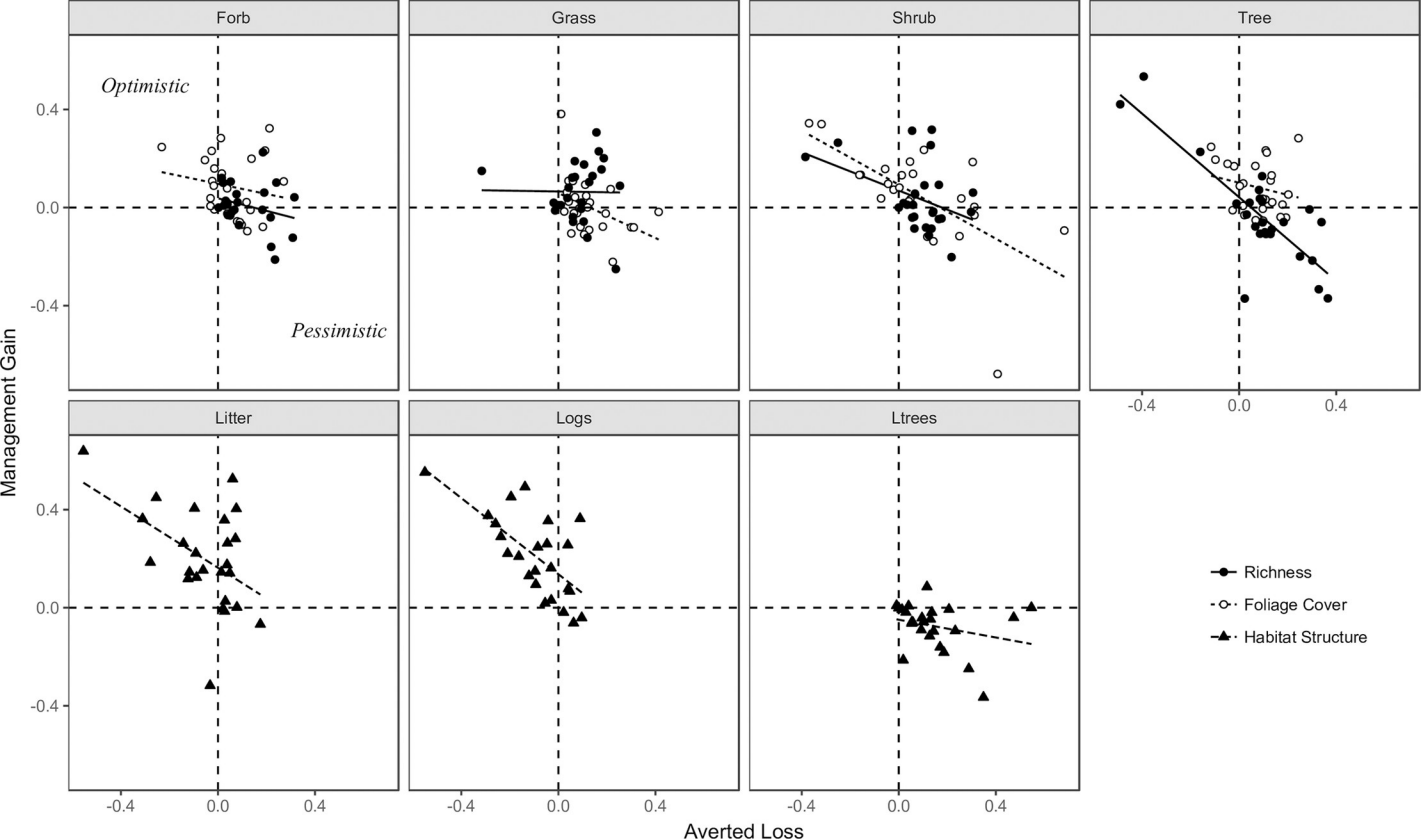
Individual expert average estimates of averted loss and management gain for each of 11 vegetation attributes, with relationships indicated by simple linear fits. Each point represents the mean opinion of an individual expert, derived from their elicited probability distributions. Estimates in the top left quadrant of each panel indicate an optimistic opinion about future trends under both BAU and an offset (i.e. low AL, high MG). Estimates in the bottom right quadrat indicate those where experts were pessimistic about future trends, regardless of management scenario (i.e. high AL, low MG). Those in the top right were pessimistic about trends under BAU but optimistic about trends with an offset (i.e. high AL, high MG).

Variation among individual experts was large, although most experts tended to be pessimistic about the future trends for large trees and optimistic about trends for logs and litter (Fig 4), regardless of the management scenario. There was some tendency to suggest that those experts who were pessimistic about BAU trends also tended to be pessimistic with an offset (Fig 4). This pattern was evident as a negative correlation between expert average estimates of averted loss and management gain, and was most apparent for logs, shrub foliage cover and tree species richness. Although some experts demonstrated a systematic bias (either consistently optimistic or pessimistic), most were not and their opinions differed among vegetation attributes (S7 File).

#### Do management gains vary according to attribute and site starting conditions?

The distribution of management gains for each attribute were explored across three WSGW scenarios. The three scenarios had different initial attribute values, and were generally thought to represent good-, moderate- and poor-quality sites (Table 2). These broad site quality descriptions were supported by predictions of aggregate vegetation condition, out of 100, derived from an ensemble of expert BRT models (see S8 File). We examined how MG varied depending on the initial condition (*BC*_*start*_) of each vegetation attribute (initial value relative to the reference value) and whether consistent patterns were evident in each of the three WSGW vegetation patches.

**Fig 5:**
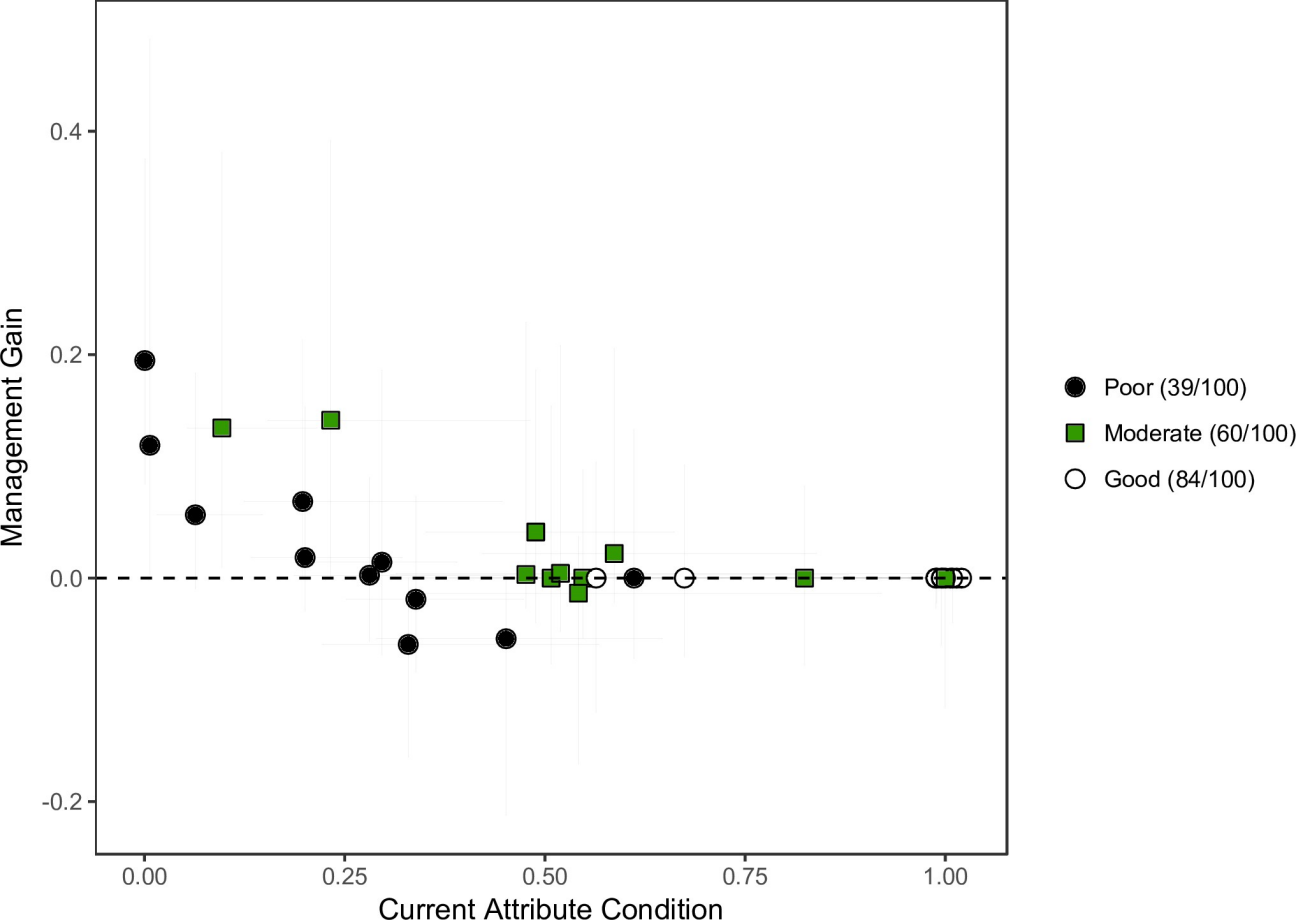
Median expert predictions of management gain and current condition for each of 11 vegetation attributes in poor-, moderate- and good-quality WSGW. Management gains are changes in the individual attributes, relative to a reference value, predicted to occur as a result of 20 years of offset management. Points are medians and lines are upper and lower quartiles derived from 2100 randomisations across 25 expert probability distributions. The broad qualitative site quality is supported by numeric estimates of aggregate vegetation condition, out of 100, derived from expert BRT models (condition score in legend in brackets).

There was generally a negative relationship between the median expert estimate of MG and the attribute’s starting value relative to the reference value (Fig 5) and attributes whose starting condition were <0.3 tended to increase with adoption of an offset. This same trend was observed in elicitations for other vegetation types (see S2 File). While the negative relationship was observed in both the poor- and moderate-quality scenarios it was most evident in the former with both positive and negative median estimates of MG, dependent on attribute starting condition. In the poor-quality scenario highest median MG were for shrub richness and cover, although logs and litter cover were also positive, while median MG for large trees and tree richness were both negative.

In the good-quality scenario the median management gain was 0 for all attributes. With the exception of logs and litter cover, median initial condition was 1, indicating that 50% of the random samples were less than the starting attribute values. In such circumstances standardisation to a reference can obscure changes on the original measurement scale, especially if experts predicted an increase following adoption of an offset. However, median estimates of absolute changes on the original scale were small and never positive (S9 File).

## Discussion

Biodiversity offsetting has been widely adopted but we often lack adequate empirical data and models upon which to make predictions of future biodiversity outcomes. Predicting the likely ecological changes with and without offsets is fundamental to offsetting, and these predictions can have significant and very real biodiversity impacts. Despite this, making such predictions remains a major technical challenge [2] and a better understanding of the uncertainty around future conservation benefits is vitally important. Here, 29 ecological professionals were asked, using two separate approaches, to make predictions about future changes in vegetation composition and structure, as a surrogate for biodiversity, with and without the adoption of a biodiversity offset. The results revealed that experts on average expected averted loss, management gain and total benefits to be modest. Individual expert predictions were often highly uncertain, and this was compounded by variation among experts. Strikingly, our analyses uncovered the perception that declines in some vegetation condition attributes were also likely at offset sites, even with the adoption of conservation management. This finding necessitates approaches that accept that some offsets will fail to achieve estimated gains, and some will deliver beyond expectation. Reasonable outcomes can be achieved overall (i.e. across many offset scenarios), but only if risks are managed appropriately. The opportunity therefore exists to minimise risk and maximise biodiversity outcomes through the identification and avoidance of high-risk offset sites.

The modest estimates of total benefits suggest that offset schemes should adopt a precautionary approach and avoid optimistic estimates of either averted loss or management gain. Several authors have argued that offset schemes often rely on unrealistically high estimates of background rates of loss (i.e. averted loss) to meet NNL requirements [8, 17]. If actual background rates of loss are indeed less than predicted, then to meet NNL these offset schemes would need to increase their reliance on management gains, achieved through conservation actions that improve on current biodiversity values. This could shift emphasis in offset site selection from high quality sites where there is much to lose to low quality sites where the potential for improvement is greater. Prediction of management gains at offset sites is, however, also inherently risky, as revealed by the substantial variation within and among experts.

One approach to managing the uncertainty of future conservation benefits is to limit trading to only those offsets that have already generated benefits [5]. The range of opinion and uncertainty described in this study lends support to the pursuit of policy and governance mechanisms that favour early or prior conservation benefits. However, the creation of prior benefits requires significant planning and investment and in practice even the often-cited wetland mitigation banks in the USA rarely provide full prior compensation [43]. In most cases, offsetting is likely to continue to rely on future benefits in which case it is imperative that monitoring and research focus on improving empirical data and models to better predict outcomes and reduce uncertainty around these predictions.

### When and where do experts believe conservation management will produce benefits?

Although predicted benefits were small, the results from both elicitation methods suggest that experts believe that management actions to address threats and pressures can often be sufficient to negate counterfactual losses. However, the study highlights that expert predicted benefits are not likely to be equivalent among all vegetation attributes. This offers opportunities for policy makers to refine offset schemes but also highlights important considerations for monitoring and evaluating current offset outcomes.

Point estimates of future aggregate biodiversity value suggested that experts believed management gains (MG) were most likely to balance counterfactual averted losses (AL) when sites were initially of moderate to low value, relative to a reference condition. Likewise, the future probability distributions provided by experts of individual attributes suggested that positive MGs would most likely arise when attributes were initially low relative to their reference. Where attributes are already at or near reference values, for example in good-quality remnant vegetation, conservation benefits are only likely if AL is relatively large and management can maintain current values. If those sites support attributes that are expected to decline, even with conservation management in place (e.g. large trees), then total benefits will inevitably be zero (or less). This would suggest that, if management gain is the objective, then offsets should be established in areas of low to moderate condition with some potential to improve.

Offsets are usually framed around the concept of NNL or Net Gain. The uncertainty surrounding the potential for gains, highlighted in the results we present here, emphasises the risks associated with loss of good-quality habitats. In most jurisdictions offsets are applied to residual impacts as part of the mitigation hierarchy, with priority given to avoidance and minimisation of impact, especially in the case of rare or high-quality habitats [44]. Despite this, situations do arise when high-quality habitat are impacted by human activities. If low to moderate condition habitats are selected as offsets because of their greater capacity for generating gains, this could lead to trading good-quality high-value habitat for larger areas of low-quality habitat, albeit habitat that has considerable potential to improve. However, this assumes that the loss of biodiversity in good-quality habitat is equivalent to a numerically similar improvement in low- or moderate-quality habitat, which need not be the case. While this can be partly addressed by nuances in the metrics (e.g. multipliers applied to rare habitats) or policy settings (e.g. regulations to prohibit clearing of rare or high-value habitats), ultimately, these decisions come down to human preferences for balance between habitat quality and extent in conservation areas.

In the WSGW scenario examined, experts consistently predicted that large trees would decline under both BAU and offset management scenarios. Apparently, experts believe that those factors that lead to declines in large trees cannot be mitigated using site-based conservation management. Large trees are slow to develop, thought to contribute disproportionately to habitat in many vegetation types [45] and have been recognised as being a keystone habitat structure [46]. Recognition that some habitat features cannot be replaced in the short- to medium-term, or their loss avoided through site-based management actions, highlights the need to avoid clearing habitats where they occur. Use of disaggregated metrics in offset calculations [14] will be important in helping identify and audit the outcomes for such habitat attributes, relative to NNL objectives.

Another key issue for managers and policy makers is determining whether the magnitude and uncertainty of management gains is likely to differ among ecosystems and vegetation types. Such knowledge could be used to develop offset metrics and policies that are sensitive to differences among ecosystems. Studies of ecological succession do suggest that there may be general differences in trajectories of recovery of vegetation along broad climatic gradients [47]. While the elicitation we presented here was primarily focused on a single ecosystem (a grassy woodland), opinions were also sought regarding four vegetation types that differ in vegetation structure and composition and occur in contrasting biomes (see S2 File). In this case there was little evidence to suggest that expert estimates of management gain consistently differed among vegetation types. Whether this result is specific to the vegetation types selected is unknown and further elicitations across a larger range of vegetation types and scenarios, coupled with empirical monitoring, is required.

### 20-year time horizon

In all exercises the expert predictions were limited to a 20-year time horizon. Yet, was this timeframe too short to observe improvements in vegetation owing to conservation management and could this have contributed to the modest averted loss and management gain estimates? Our choice of 20 years aligns with the Australian Commonwealth and New South Wales offset policies [13, 33]; is a reasonable length of time to accrue many habitat attributes; is at the outer limit of any plausible attempt to predict biodiversity outcomes; and is within the working knowledge of many of the experts (see S1 File). Timeframes greater than 20 years would have required most experts to predict to horizons greater than their working experience. In the context of biological processes, losses or gains of habitat over longer timeframes could necessitate consideration of temporal equivalency (i.e. the discounting of future gains)[48], further complicating the elicitation process. Estimates across a range of timeframes would however be useful to better identify those attributes thought to respond rapidly and those for which long-term monitoring will be necessary. While 20 years is a reasonable expectation of long-term monitoring programs, monitoring and evaluation of biodiversity offsets could also demand detection of early trends, for example within 5–10 years.

### The potential role for expert elicitation in offset calculations

There is increasing expectation that offset calculations should incorporate appropriate estimates of uncertainty [6]. Yet while there are offset schemes that explicitly modify predicted conservation benefits by estimates of uncertainty, there is little guidance for how these predictions should be obtained [6, 15]. Inevitably empirical data will often be scarce and those tasked with identifying and assessing offsets may need to rely heavily on opinion. When timeframes are short, and resources limited there may be a tendency to base estimates on the opinions of few experts or use unstructured, informal methods of elicitation.

Structured, transparent elicitation of opinion, capable of adequately describing underlying uncertainty are necessary to counter bias and improve reliability and confidence in the outcome [49]. Unless data are obtained from multiple experts and in a structured manner there may be little confidence that offset obligations adequately reflect opinion, even when uncertainty is considered. It is well recognised that experts and non-experts tend to be overly precise and overconfident [50], which could result in overestimation of the likelihood of the outcome. We have clearly shown that expert opinions can be divergent and biased estimates could easily result if opinions are sought from too few individuals or a limited pool of experts.

Even with carefully structured elicitation methods it can be difficult to guard against all forms of bias and overconfidence. For example, the belief that environmental conditions will worsen in the future, so called temporal pessimism, is thought to be widespread [51], but has been shown to differ among professions [52]. If present among our experts, it could result in an overestimation of the likely losses under a BAU scenario (large estimates of AL) and underestimation of potential improvement with a biodiversity offset (low estimates of MG). A key underlying assumption of most offset schemes is that conditions will worsen under a BAU scenario but will improve upon adoption of an offset. If our experts held this view, then they should be consistently pessimistic about a BAU scenario but optimistic when conservation management actions were adopted. We instead found that, for a given attribute, experts who were more pessimistic about the counterfactual were also often less inclined to believe, on average, relative values would improve with an offset. While, most experts were not systematically optimistic or pessimistic, a small number were. Most importantly some of those with a systematic bias also held completely opposing opinions (S7 File). This is compelling and indicates that these experts may have vastly different experiences and understanding of the temporal dynamics of grassy woodlands. Unfortunately, we lack the objective probability distributions of the effects of biodiversity offsetting that would be necessary to weight these different responses. However, a better understanding of the source of these conflicting opinions could also improve the applicability of the elicited data.

Overconfidence, the tendency to provide narrow, overly precise estimates [53], is pervasive in most forms of expert elicitation [50]. Overconfidence could lead to unjustified optimism or pessimism about the potential of offsets to provide adequate compensation for biodiversity losses. While our first elicitation approach did not attempt to capture uncertainty, the second did and in this instance, we guarded against overconfidence in two main ways. Firstly, we used the trial roulette method with a graphical interface which along with similar methods (e.g. Subjective Probability Interval Estimates, [54]) has been found to substantially reduce expert overconfidence [34, 54]. Secondly, experts were required to complete five training exercises, with feedback. During feedback experts were encouraged to consider whether their specified range captured the observed value. While we cannot test whether overconfidence was completely mitigated against, our approach to combining predictions took full account of each expert's subjective probability distribution and hence the median and mean estimates incorporate both inter- and intra- expert variation.

Our first elicitation, based on single-point best-guess estimates, could be prone to overconfidence. While we can estimate variation among experts, individual predictions are treated as precise and are not influenced by inherent within-expert uncertainty. However, while this approach will not have fully captured the range of variation, it is notable that the general trends are consistent with those of the more comprehensive trial roulette approach. Further, this exercise was simple and could be quickly and easily completed; at each workshop this exercise was completed within 30 minutes, including explanatory discussion and feedback. While eliciting full subjective probability distributions may be ideal, there is also a case for balancing overconfidence, knowledge of individual expert uncertainty and better understanding of the range of opinion among experts.

## Conclusions

Biodiversity offsetting faces significant ethical, social, technical and governance challenges [2]. Our structured expert elicitation does not resolve these issues but has addressed a key area of uncertainty in biodiversity offsetting—the prediction of future changes in vegetation composition and structural attributes, that generally provide resources and habitat for biodiversity. While expert opinion should not replace good empirical data and ecological models, expert predictions can be complementary and provide useful guidance for prudent offsetting policy development.

In the case-studies presented here, average expert predictions of conservation benefits were small and individual opinions were often divergent. This suggests a general perception that biodiversity offsetting, based on adoption of conservation management actions to avert loss and improve current values, is inherently risky. It highlights an urgent need to better identify those components of biodiversity most likely to benefit from conservation management and understand those situations when offset obligations are most likely to be met. Given the global proliferation of biodiversity offsetting there is now a strong imperative to monitor and evaluate existing offset sites to determine whether the magnitude and range of predicted biodiversity outcomes can be realised.

## Supporting information

S1 File Selection of experts, online survey & questionnaire for potential participants and summary of survey results. (PDF)

S2 File Management gains, do they vary according to vegetation type?. (PDF)

S3 File Elicitation material and scenarios. (PDF)

S4 File Fitted boosted regression tree functions for initial aggregate vegetation condition, management gain, averted loss and total benefit. (PDF)

S5 File Boxplots of individual expert estimates of aggregate management gain. (PDF)

S6 File Boxplots of individual expert probability distributions of MG, AL and TB. (PDF)

S7 File Are individual experts consistently optimistic or pessimistic? Individual expert mean estimates of averted loss and management gain. (PDF)

S8 File Boosted regression tree for predicting vegetation condition to new sites. (PDF)

S9 File Predicted change in individual attribute values with offset on original measurement scale. (PDF)
