# Supplementary material for: Expert predictions of changes in vegetation condition reveal perceived risks in biodiversity offsetting
Source: PLoS One. 2019 May 8;14(5):e0216703. doi: 10.1371/journal.pone.0216703 (PMC6505952; doi:10.1371/journal.pone.0216703)
Supplement: S1 File — (PDF) [file pone.0216703.s001.pdf]

S1 Selection of experts, online survey & questionnaire for potential participants and summary of survey results

A pool of 94 native vegetation professionals from across south-eastern Australia was compiled through searches of published literature on vegetation dynamics and monitoring, and from native vegetation management practitioners and researchers known to the authors and colleagues (see acknowledgements in main manuscript). The pool included academics, native vegetation managers, restoration practitioners, government scientists and private environmental consultants.

Experts were ranked based on breadth of experience (assessed by the authors), on whether their professional work covered multiple vegetation types or regions and whether they were known to have undertaken temporal vegetation monitoring. The top-ranked thirty-nine experts were invited by email to complete an online questionnaire and asked to participate in one of three two-day workshops. Experts were offered funding to cover time and all expenses involved with the workshops. A total of 29 experts attended one of four 1 to 1.5-day workshops.

The online questionnaire asked respondents to self-rate their experience in native vegetation management, plant and restoration ecology, vegetation field survey and monitoring. Experts were also asked to nominate those biogeographic zones and vegetation types where they have undertaken work (see below). Thirty-seven experts completed the online survey.

Most experts claimed to have experience in the monitoring of native vegetation. While a range of methods were used, most had undertaken plot based longitudinal monitoring which involved repeat observations from the same sets of plots.

Online questionnaire

<u>Question 1.</u>

Please rate the extent to which you agree with the following statements using the scale “Strongly Disagree”, “Disagree”, "Neither agree nor disagree”, “Agree”, “Strongly Agree”:

I have expertise in plant ecology

I have expertise in restoration ecology

I have expertise in the management of native vegetation

I have expertise in the monitoring of native vegetation

I have experience in grassland ecology and/or management

I have experience in grassy woodland ecology and/or management

I have experience in dry forest ecology and/or management

I have experience in forested wetland ecology and/or management

I have experience in rainforest ecology and/or management

I have experience in wet forest ecology and/or management

I have experience in heathland ecology and/or management

I have experience in freshwater wetland ecology and/or management

I have experience in saline wetland ecology and/or management

I have experience in semi-arid woodland ecology and/or management

I have experience in arid shrubland ecology and/or management

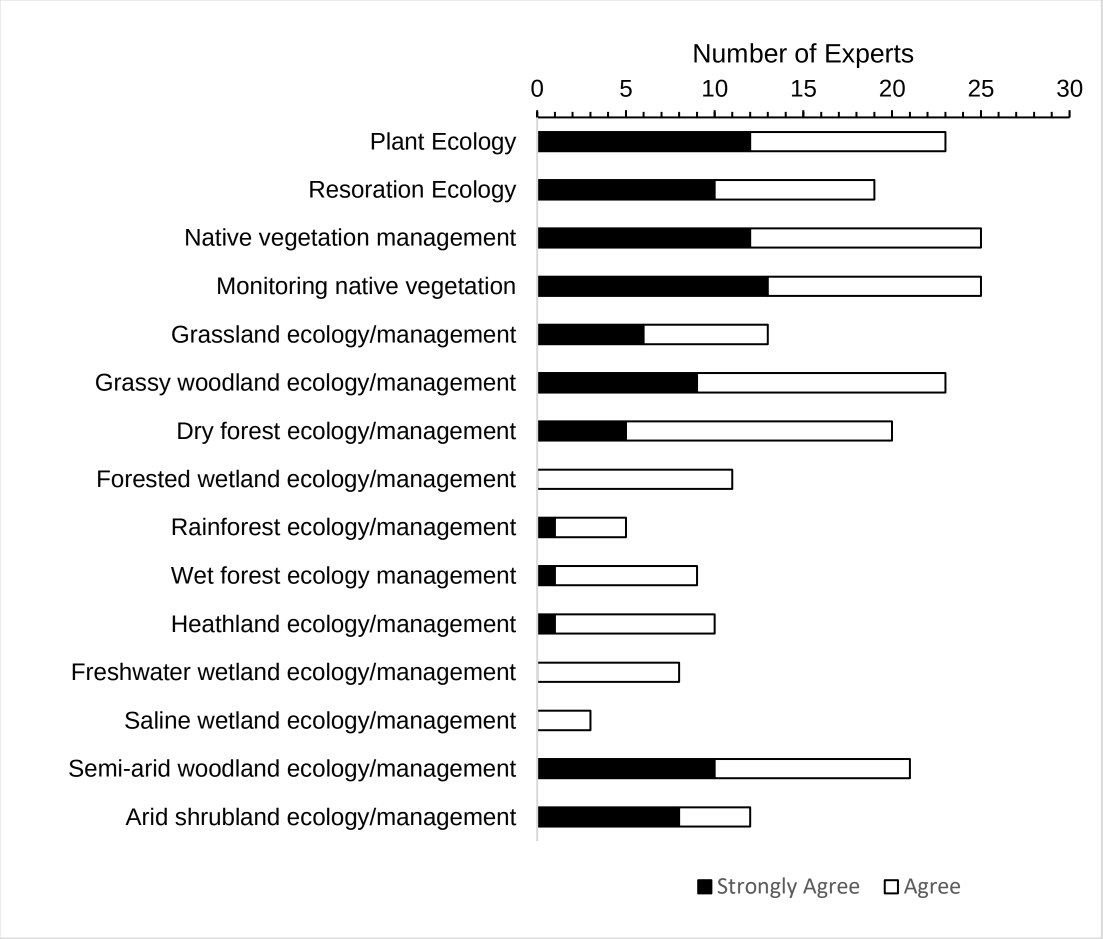

Figure S1.1 The number of experts who self-rated "agree" or "strongly agree" to statements describing them as an expert in various aspects of native vegetation ecology or management. Alternative responses available to experts, the results of which are not shown, were "neither agree or disagree", "disagree" and "strongly disagree".

Question 2

Have you undertaken research or monitoring designed to understand the temporal dynamics of native vegetation? You may provide more than one answer if applicable.
Answer Options

1. Yes, formal plot based longitudinal monitoring/research
2. Yes, informal visual observations
3. Yes, based on spatially separated plots using a space-for-time method
4. Yes, other (please briefly describe in comments field below)
5. No, my experience is from one-off spatially separated plot-based surveys, not focused on a chronosequence
6. No

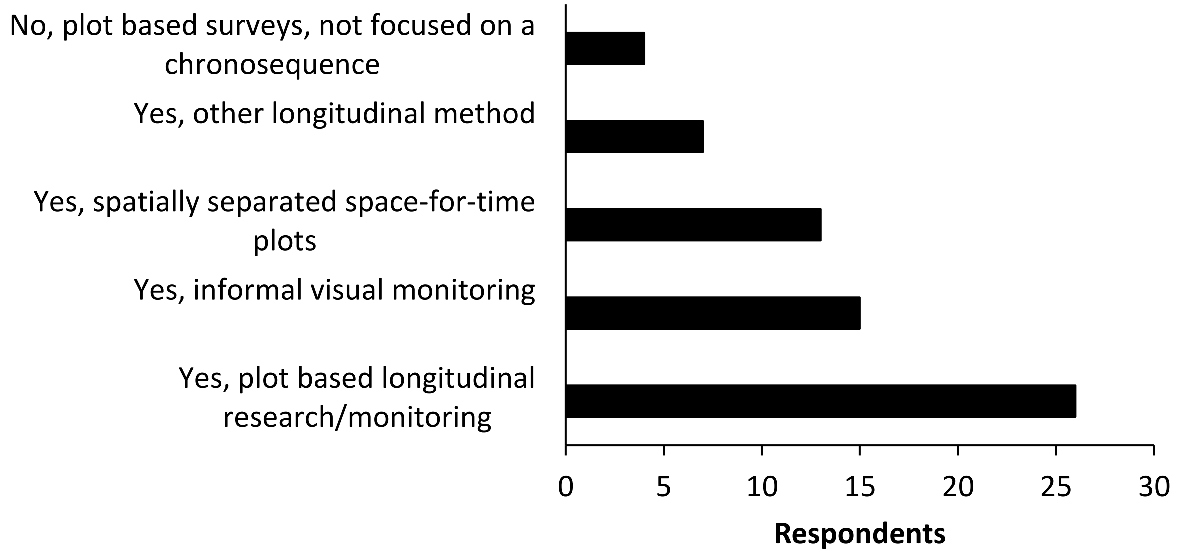

Figure S1.2 Experts self-rated experience undertaking research or monitoring designed to understand the temporal dynamics of native vegetation.

Question 3

If you answered yes to Question 2, what is the maximum duration of these observations (in years)?
Answer Options
1. up to 3 years<br>2. 3-5 years<br>3. 5-10 years<br>4. 10-15 years<br>5. 15-20 years<br>6. >20 years

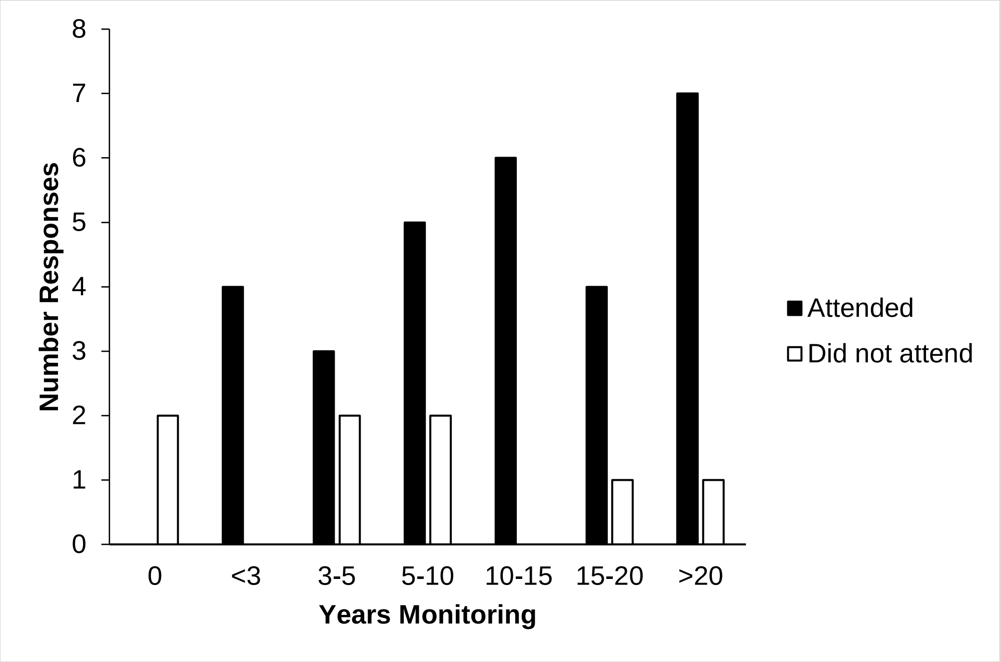

Figure S1.3 Maximum duration of monitoring designed to understand the temporal dynamics of native vegetation for each of 37 experts, 29 of whom attended an elicitation workshop. Of the 29 experts attending the workshops, 17 were government employees (ecological researchers, botanists or land managers employed in either NSW, Victoria, Queensland or Federally), 6 were university academics, 5 were privately employed as consultants and one worked for a non-government organisation.

Question 4

If you answered yes to question 2, please select those vegetation formations within which the research/monitoring applied (you may select more than one)
Answer Options
Grassland
Grassy Woodland
Semi-arid Woodland
Arid Shrubland
Dry forest
Wet forest
Rainforest
Freshwater wetland
Saline wetland
Heathland

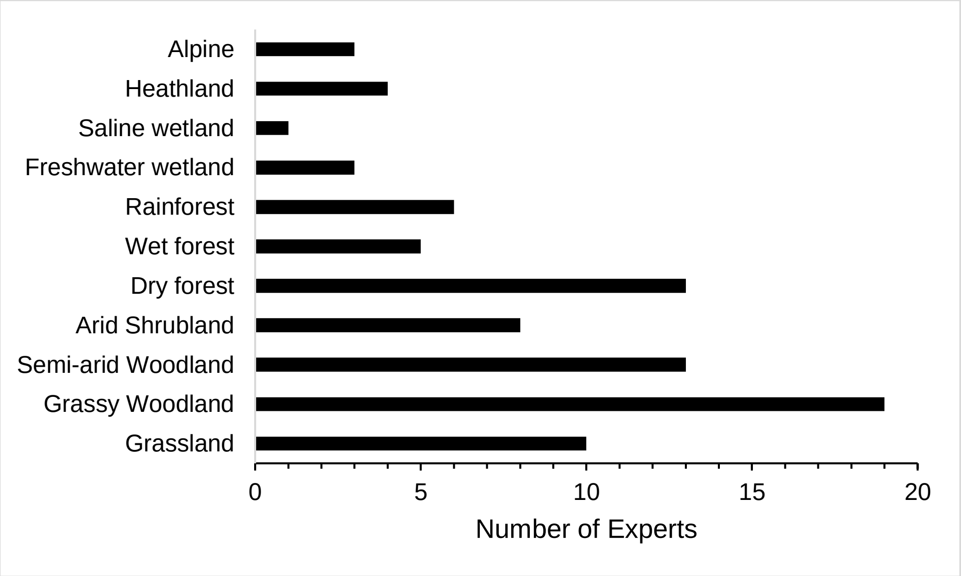

Figure S1.4 The number of experts with experience undertaking vegetation monitoring in each of 11 vegetation formations
