# Supplementary material for: Expert predictions of changes in vegetation condition reveal perceived risks in biodiversity offsetting
Source: PLoS One. 2019 May 8;14(5):e0216703. doi: 10.1371/journal.pone.0216703 (PMC6505952; doi:10.1371/journal.pone.0216703)
Supplement: S2 File — (PDF) [file pone.0216703.s002.pdf]

S2 Management gains, do they vary according to vegetation type?

The distribution of individual attribute MG was compared among four different vegetation types. These occurred along a gradient of mean annual rainfall and annual NPP (Table S2.1). We examined how MG varied depending on the initial condition (BC_{start}) of each vegetation attribute (initial value relative to the reference value) and whether consistent patterns were evident in each of the four vegetation types.

Table S2.1. The four vegetation types for which future values of 13 vegetation attributes were elicited. Rainfall is the median long-term annual. NPP is median annual net primary productivity (g C/m^2) estimated from 2000 and 2015 MODIS 17A3 annual Net Primary Productivity data [1].

Scenario	Vegetation Class	Bioregion	NPP	Rain (mm)
1	Sand Plain Mulga Shrubland	Mulga Lands	141	225
2	Inland Floodplain Woodland	Riverina	255	385
3	Western Slopes Grassy Woodland	Brigalow Belt South	574	590
4	Sub-tropical rainforest	NSW North Coast	1685	1430

Results suggested that the experts believed that there was likely to be a negative relationship between MG and an attribute's initial condition (Fig S3.1). An improvement in the relative value of an attribute, following adoption of an offset, was predicted when the attribute starting value was approximately less than 50% of the reference value. All vegetation types had a similar spread of initial attribute conditions, although no attributes had a median condition <0.3 within the Inland Floodplain Woodland in the Riverina.

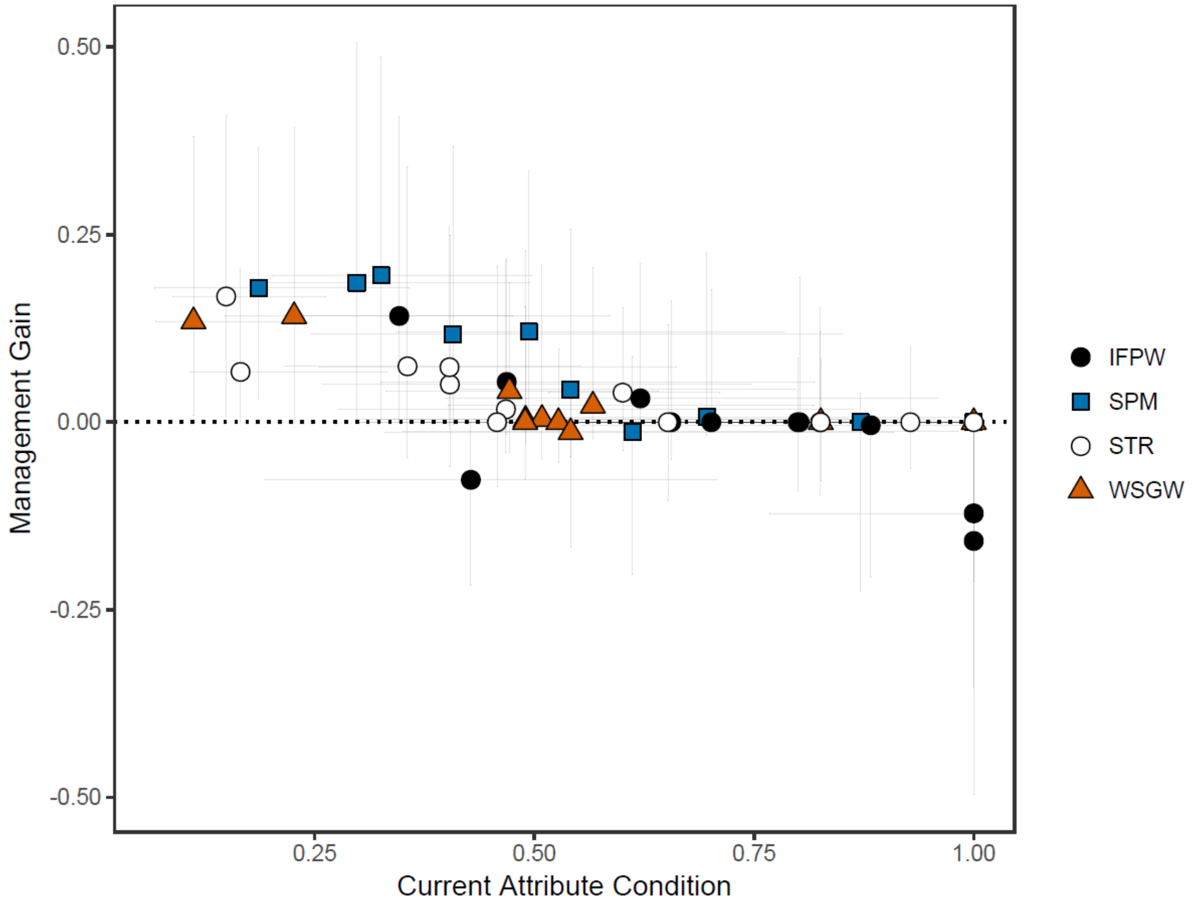

Fig S2.1. Expert predictions of management gains and current condition for each of 13 vegetation attributes in four different vegetation types, whose starting conditions were selected to approximate a vegetation patch typical of a grazed moderate condition. Fern richness and foliage cover were only included for Sub-tropical rainforest and large trees have been excluded for Sand plain mulga shrubland. IFPW – Inland floodplain woodland, SPM = Sand plain mulga shrubland, STR = Sub-tropical rainforest, WSGW = Western slopes grassy woodland.

<u>References</u>

1. Running SW, Nemani RR, Heinsch FA, Zhao MS, Reeves M, Hashimoto H. A continuous satellite-derived measure of global terrestrial primary production. Bioscience. 2004; 54: 547-60. doi: Doi 10.1641/0006-3568(2004)054[0547:Acsmog]2.0.Co;2.
