# Supplementary material for: Expert predictions of changes in vegetation condition reveal perceived risks in biodiversity offsetting
Source: PLoS One. 2019 May 8;14(5):e0216703. doi: 10.1371/journal.pone.0216703 (PMC6505952; doi:10.1371/journal.pone.0216703)

S3 Elicitation material and scenarios

A. Example of synthetic site for aggregate vegetation condition

DESCRIPTION

Landscape Native Vegetation Cover	**70%**
Invasive alien plant cover	**30%**
Alien plant ground cover	**40%**

Dominant Species

Trees	*Eucalyptus albens*
Shrubs	*Pimelea neo-anglica*
Grass & grass-like cover	*Hyparrhenia hirta*, Austrostipa scabra, Aristida personata, Chloris truncata*
Forbs	*Dichondra repens, Sida corrugata, Vittadinia cuneata*
Ferns	*Cheilanthes sieberi*

Attribute	***Start Value***	***Attribute***	***Start Value***
Tree cover (%, crown cover)	15	Tree richness (no. spp)	1
Shrub cover (%, crown cover)	5	Shrub richness (no. spp)	2
Grass & grass-like cover (%, crown cover)	36	Grass & grass-like richness (no. spp)	5
Forb cover (%, crown cover)	7	Forb richness (no. spp)	9
Fern cover (%, crown cover)	0.5	Fern richness (no. spp)	1
Number of large trees/0.1 ha	3	Length woody debris >10cm diameter (m)	2
Litter cover (%)	5		

*Invasive alien plant species

B. Practice Exercises

To familiarise experts with the trial histogram method, provide them with confidence in the task and a base level of calibration, each expert initially undertook five practice exercises derived from published studies. These exercises were not intended to facilitate convergence in the actual elicitation, nor were they intended to be used to weight individual experts. Each of the exercises focused on quantities of relevance with actual measured changes over a specific time interval: changes in the diameter at breast height of three different tree species after 20 years (Ngugi et al 2015), changes in alpine shrub foliage cover after 20 years of grazing exclusion (Scherrer and Pickering 2005) and changes in total native ground cover in a floodplain grassy woodland after 5 years of grazing exclusion (Lunt et al 2007). In each case experts were given a description from the publication, including a starting value and were asked to estimate a future value at the end of the fixed time horizon. After each exercise the mean, and if available an estimate of variation, drawn from the relevant publication were presented to the group. Discussion among group members was encouraged after each elicitation and experts were asked to consider how choice of θ_{min}, θ_{max} and allocation of chips into bins affected their ability to capture the range of observed future values within their own elicited probability distributions, particularly when they were estimating uncertain quantities.

Ngugi MR, Doley D, Cant M, Botkin DB. Growth rates of Eucalyptus and other Australian native tree species derived from seven decades of growth monitoring. J Forestry Res. 2015; 26: 811-26. doi: 10.1007/s11676-015-0095-z.

Scherrer P, Pickering CM. Recovery of alpine vegetation from grazing and drought: Data from long-term photoquadrats in Kosciuszko National Park, Australia. Arct Antarct Alp Res. 2005; 37: 574-84. doi: 10.1657/1523-0430(2005)037[0574:Roavfg]2.0.Co;2.

Lunt ID, Jansen A, Binns DL, Kenny SA. Long-term effects of exclusion of grazing stock on degraded herbaceous plant communities in a riparian Eucalyptus camaldulensis forest in south-eastern Australia. Austral Ecol. 2007; 32: 937-49. doi: 10.1111/j.1442-9993.2007.01782.x.

Practice exercises 1-3: Estimate tree diameter

The first three practice exercises required experts to generate a distribution of their estimate of the range of likely future tree diameters in 20 years given a starting tree diameter of 30mm. They were asked to complete the exercise for three different tree species, each occurring in a region with different average annual rainfall. A completed example is presented below.

1. SPECIES: *Callitris glaucophylla* STARTING DBH: 30cm AVERAGE ANNUAL RAINFALL : 600mm

**MINIMUM PLAUSIBLE VALUE**	30
**MAXIMUM PLAUSIBLE VALUE**	50

Tree Diameter	Number of trees
32	15
34	50
36	15
38	10
40	10
42	0
44	0
46	0
48	0
50	0
	**100 allocated**

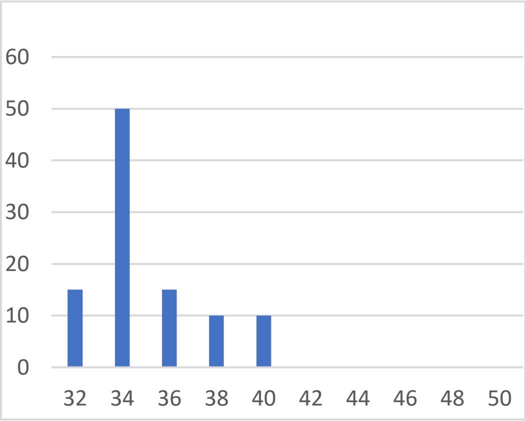

2. SPECIES: *Eucalyptus crebra* STARTING DBH: 30cm AVERAGE ANNUAL RAINFALL : 800mm

**MINIMUM PLAUSIBLE VALUE**	30
**MAXIMUM PLAUSIBLE VALUE**	55

Tree Diameter	Number of trees
32.5	0
35	5
37.5	10
40	45
42.5	20
45	15
47.5	5
50	0
52.5	0
55	0
	**100 allocated**

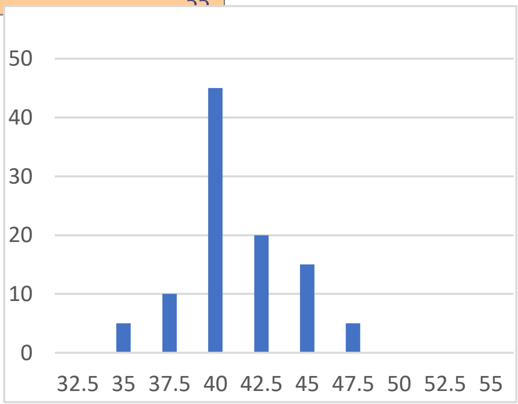

3. SPECIES: *Eucalyptus pilularis* STARTING DBH: 30cm AVERAGE ANNUAL RAINFALL : 1600mm

**MINIMUM PLAUSIBLE VALUE**	30
**MAXIMUM PLAUSIBLE VALUE**	60

Tree Diameter	Number of trees
33	0
36	0
39	0
42	0
45	5
48	20
51	45
54	25
57	5
60	0
	**100 allocated**

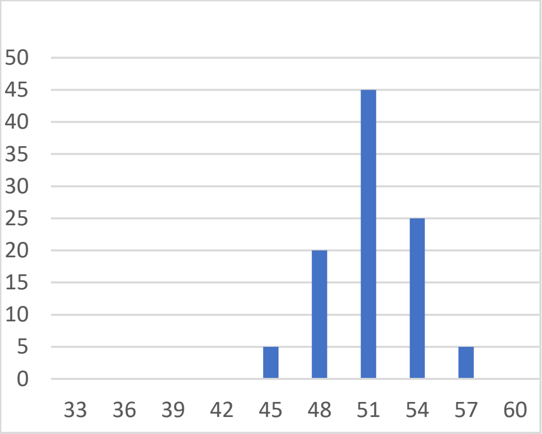

Practice exercises 4-5: Estimate Plant Cover

In the remaining two practice exercises experts were asked to estimate a distribution that reflected their opinion about future vegetation cover given a starting cover of a specific attribute (either shrub cover or total native ground cover), a prediction timeframe, a description of the vegetation, biogeographic location, management context and average annual rainfall. Completed examples are provided below.

4. Shrub cover in an alpine herb field in the Australian Alps. Previously grazed by livestock with initial estimate of shrub cover taken the year after livestock removal. Livestock excluded for 20 years. Average annual rainfall ~1800mm

Initial shrub cover: 0.1%
Prediction timeframe: 20 YEARS

**MINIMUM PLAUSIBLE VALUE**	0
**MAXIMUM PLAUSIBLE VALUE**	20

Cover (%)	Number of plots
2	15
4	20
6	30
8	20
10	7
12	5
14	2
16	1
18	0
20	0
	**100 plots allocated**

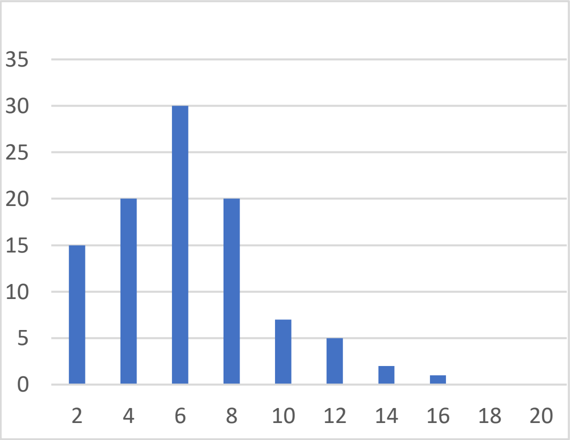

5. Native ground layer cover in Riparian River redgum (*Eucalyptus camaldulensis*) open forest in the NSW Riverina bioregion. State forest with history of livestock grazing. Initial estimates of native ground cover in the same year as cattle excluded. Area dominated by exotic annuals (60% ground cover). Average annual rainfall 425mm (yr0=360mm, yr1=340mm, yr2=570mm, yr3=580mm, yr4=240mm, yr5=460mm)

Initial native ground cover : 5%
Prediction timeframe: 5 years

**MINIMUM PLAUSIBLE VALUE**	0
**MAXIMUM PLAUSIBLE VALUE**	30

Cover (%)	Number of plots
3	5
6	20
9	35
12	20
15	10
18	5
21	3
24	1
27	1
30	0
	**100 plots allocated**

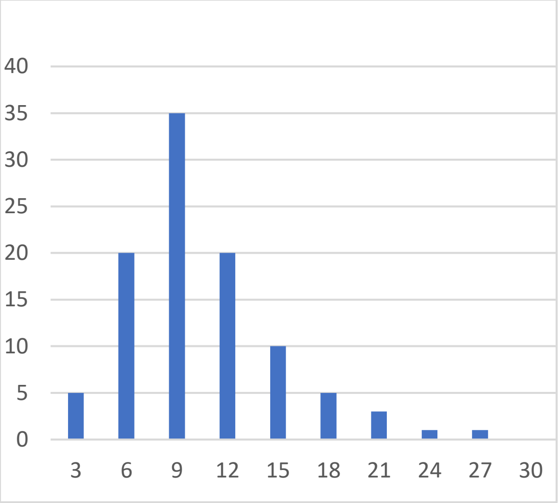

C. **Site descriptions and starting values for each of the three Western Slope Grassy Woodlands scenarios prepared for the trial roulette elicitation**

SCENARIO 1

DESCRIPTION

Vegetation Class	**Western Slopes Grassy Woodland**
IBRA region	**Brigalow Belt South**
Soil/ landscape description	**Clay loam (red brown earths) on footslopes**
Landscape Native Vegetation Cover	**70%**
**Rainfall**	
10th %ile	395
Median	590
90th %ile	800
**Dominant Native Species**	
Trees	*Eucalyptus albens*
Shrubs	*Pimelea neo-anglica*
Grass & grass-like cover	*Austrostipa scabra, Aristida ramosa, Chloris truncata*
Forbs	*Dichondra repens, Sida corrugata, Vittadinia cuneata*
Ferns	*Cheilanthes sieberi*
Total alien plant cover	**10%**

Attribute	*Start Value*	*Attribute*	*Start Value*
**Tree cover (%, crown cover)**	**15**	**Tree richness (no. spp)**	**1**
**Shrub cover (%, crown cover)**	**5**	**Shrub richness (no. spp)**	**2**
**Grass & grass-like cover (%, crown cover)**	**36**	**Grass & grass-like richness (no. spp)**	**5**
**Forb cover (%, crown cover)**	**7**	**Forb richness (no. spp)**	**9**
**Fern cover (%, crown cover)**	**0.50**	**Fern richness (no. spp)**	**1**
**Number of large trees/0.1 ha**	**3**		
**Litter cover (%)**	**5**		
**Length woody debris >10cm diameter (m)**	**2**		

SCENARIO 2

DESCRIPTION

Vegetation Class	**Western Slopes Grassy Woodland**
IBRA region	**Brigalow Belt South**
Soil/ landscape description	**Clay loam (red brown earths) on footslopes**
Landscape Native Vegetation Cover	**70%**
**Rainfall**	
10th %ile	395
Median	590
90th %ile	800
**Dominant Native Species**	
Trees	*Eucalyptus albens, E. melanophloia*
Shrubs	*Eromophila debilis, Pimelea neo-anglica*
Grass & grass-like cover	*Aristida ramosa, Lomandra multiflora, Sorghum leiocladum, Themeda triandra*
Forbs	*Dianella revoluta, Geranium solanderi, Calotis lappulacea, Desmodium brachypodum*
Ferns	*Cheilanthes sieberi*
Total alien plant cover	**10%**

Attribute	*Start Value*	*Attribute*	*Start Value*
**Tree cover (%, crown cover)**	**34**	**Tree richness (no. spp)**	**3**
**Shrub cover (%, crown cover)**	**15**	**Shrub richness (no. spp)**	**5**
**Grass & grass-like cover (%, crown cover)**	**63**	**Grass & grass-like richness (no. spp)**	**14**
**Forb cover (%, crown cover)**	**18**	**Forb richness (no. spp)**	**20**
**Fern cover (%, crown cover)**	**1**	**Fern richness (no. spp)**	**1**
**Number of large trees/0.1 ha**	**6**		
**Litter cover (%)**	**15**		
**Length woody debris >10cm diameter (m)**	**10**		

SCENARIO 3 DESCRIPTION

Vegetation Class	**Western Slopes Grassy Woodland**
IBRA region	**Brigalow Belt South**
Soil/ landscape description	**Clay loam (red brown earths) on footslopes**
Landscape Native Vegetation Cover	**70%**
**Rainfall**	
10th %ile	395
Median	590
90th %ile	800
**Dominant Native Species**	
Trees	*Eucalyptus albens*
Shrubs	
Grass & grass-like cover	*Rytidosperma caespitosa, Austrostipa scabra subsp. scabra*
Forbs	*Sida corrugata, Rumex brownii*
Ferns	
Total alien plant cover	**40%**

Attribute	***Start Value***	***Attribute***	***Start Value***
**Tree cover (%, crown cover)**	**7**	**Tree richness (no. spp)**	**1**
**Shrub cover (%, crown cover)**	**0**	**Shrub richness (no. spp)**	**0**
**Grass & grass-like cover (%, crown cover)**	**26**	**Grass & grass-like richness (no. spp)**	**4**
**Forb cover (%, crown cover)**	**3**	**Forb richness (no. spp)**	**4**
**Fern cover (%, crown cover)**	**0**	**Fern richness (no. spp)**	**0**
**Number of large trees/0.1 ha**	**1**		
**Litter cover (%)**	**5**		
**Length woody debris >10cm diameter (m)**	**1**		

D. Example of spreadsheet with interactive histograms for capture of expert subjective probability distributions

What does a benchmark condition site look like?

In this first task you are required to estimate the most likely values and the range of values you would expect to observe within a western slopes grassy woodland that is in "REFERENCE" condition. A REFERENCE condition in this context refers to the ***best attainable*** condition in the ***contemporary landscape.*** Best attainable sites would be those that have not been cleared, fertilised or cultivated. Your estimates of the benchmark condition provide a useful context for your estimates in later scenarios.

Generating the distribution of possible outcomes: You are asked to imagine 100 independent reference condition 2ha patches of western slopes grassy woodland in the Brigalow Belt South bioregion. For each attribute you are to allocate these 100 sites across 10 "bins", your allocation will generate a histogram which you can use to visually confirm your allocations. Initially allocate the most "sites" to the bin that you think is the most likely and continue to allocate sites to the next most likely bin until all sites are allocated. After the initial allocation consider whether your histogram captures the range of likely values you expect to occur within benchmark condition vegetation?

You should assume that your estimates are obtained from a randomly placed 20m x 20m (or 20m x 50m for estimates of large trees amd coarse woody debris) sampling plot within a uniform 2ha patch of vegetation. The vegetation is sampled in spring (October) in a year of median annual rainfall.

**DESCRIPTION**	
Vegetation Class	**Western Slopes Grassy Woodland**
IBRA region	**Brigalow Belt South**
Soil/ landscape description	**Clay loam (red brown earths) on footslopes**

**Rainfall(mm)**	Annual
10th Percentile	390
Median	585
90th Percentile	800

1. TREE COVER

**MINIMUM PLAUSIBLE VALUE**	0
**MAXIMUM PLAUSIBLE VALUE**	50
Cover Range (upper value of bin)	Number of Sites
5	0
10	5
15	8
20	12
25	15
30	20
35	15
40	10
45	10
50	5
	100 sites allocated
Sites Allocated =	100

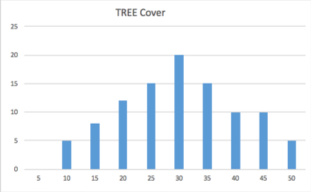

4. FORB COVER

**MINIMUM PLAUSIBLE VALUE**	0
**MAXIMUM PLAUSIBLE VALUE**	15
Cover Range (upper value of bin)	Number of Sites
1.5	5
3	10
4.5	15
6	20
7.5	18
9	12
10.5	10
12	8
13.5	2
15	0
	100 sites allocated
Sites Allocated =	100

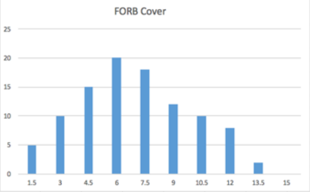

2. SHRUB COVER

**MINIMUM PLAUSIBLE VALUE**	0
**MAXIMUM PLAUSIBLE VALUE**	40
Cover Range (upper value of bin)	Number of Sites
4	5
8	10
12	15
16	20
20	18
24	12
28	10
32	8
36	2
40	0
	100 sites allocated
Sites Allocated =	100

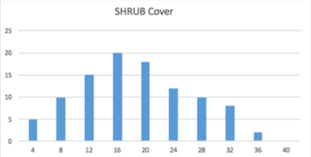

5. Fern Cover

**MINIMUM PLAUSIBLE VALUE**	0
**MAXIMUM PLAUSIBLE VALUE**	2
Cover Range (upper value of bin)	Number of Sites
0.2	10
0.4	15
0.6	20
0.8	20
1	15
1.2	10
1.4	5
1.6	3
1.8	2
2	0
	100 sites allocated
Sites Allocated =	100

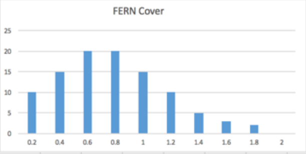

3. GRASS & GRASS-LIKE COVER

**MINIMUM PLAUSIBLE VALUE**	0
**MAXIMUM PLAUSIBLE VALUE**	70
Cover Range	Number of Sites
7	1
14	3
21	5
28	8
35	10
42	20
49	25
56	20
63	10
70	5
	ERROR, >100 sites
Sites Allocated =	107

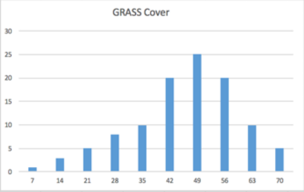

Supplement: S3 File — (PDF) [file pone.0216703.s003.pdf]
