# Supplementary material for: Expert predictions of changes in vegetation condition reveal perceived risks in biodiversity offsetting
Source: PLoS One. 2019 May 8;14(5):e0216703. doi: 10.1371/journal.pone.0216703 (PMC6505952; doi:10.1371/journal.pone.0216703)
Supplement: S4 File — (PDF) [file pone.0216703.s004.pdf]

S4 Fitted boosted regression tree functions for initial aggregate vegetation condition, management gain, averted loss and total benefit

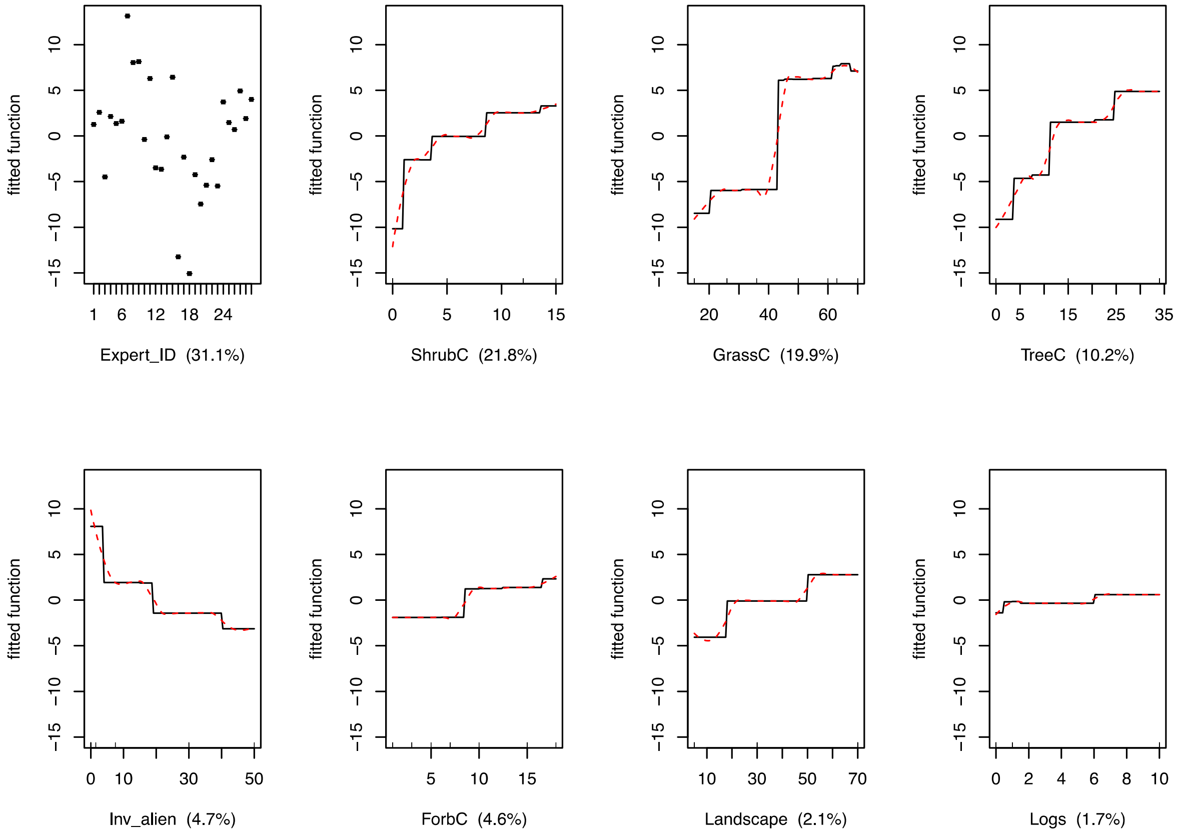

Fig S4.1 Fitted functions for expert aggregate initial condition score (y) and plot attribute values (x), for each of the 8 variables with highest normalised importance in Boosted Regression Tree models.

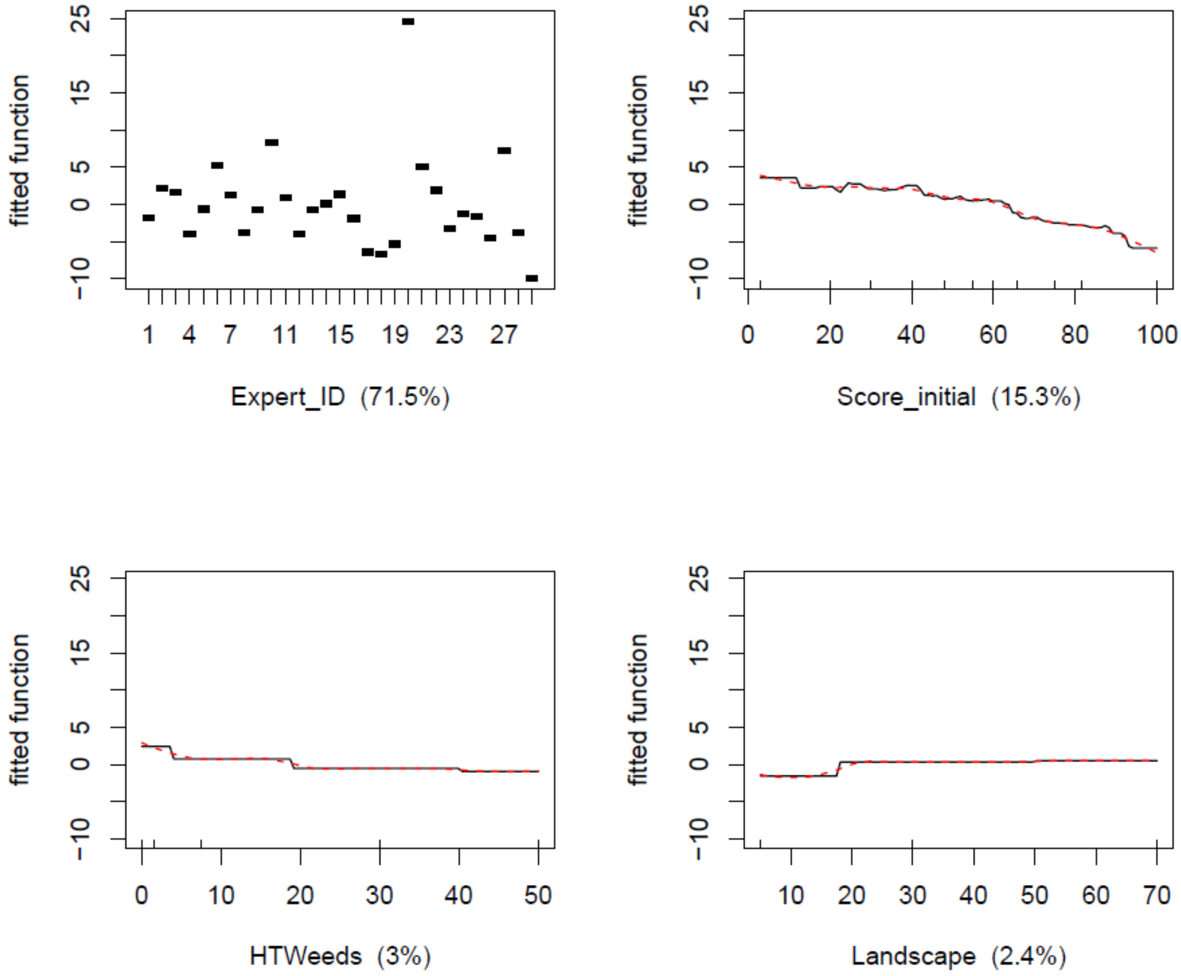

Fig S4.2 Fitted functions for expert aggregate management gain (y) and plot attribute values (x), for each of the 4 variables with highest normalised importance (expert identity, initial aggregate condition score, invasive alien plant cover and landscape vegetation cover) in Boosted Regression Tree models.

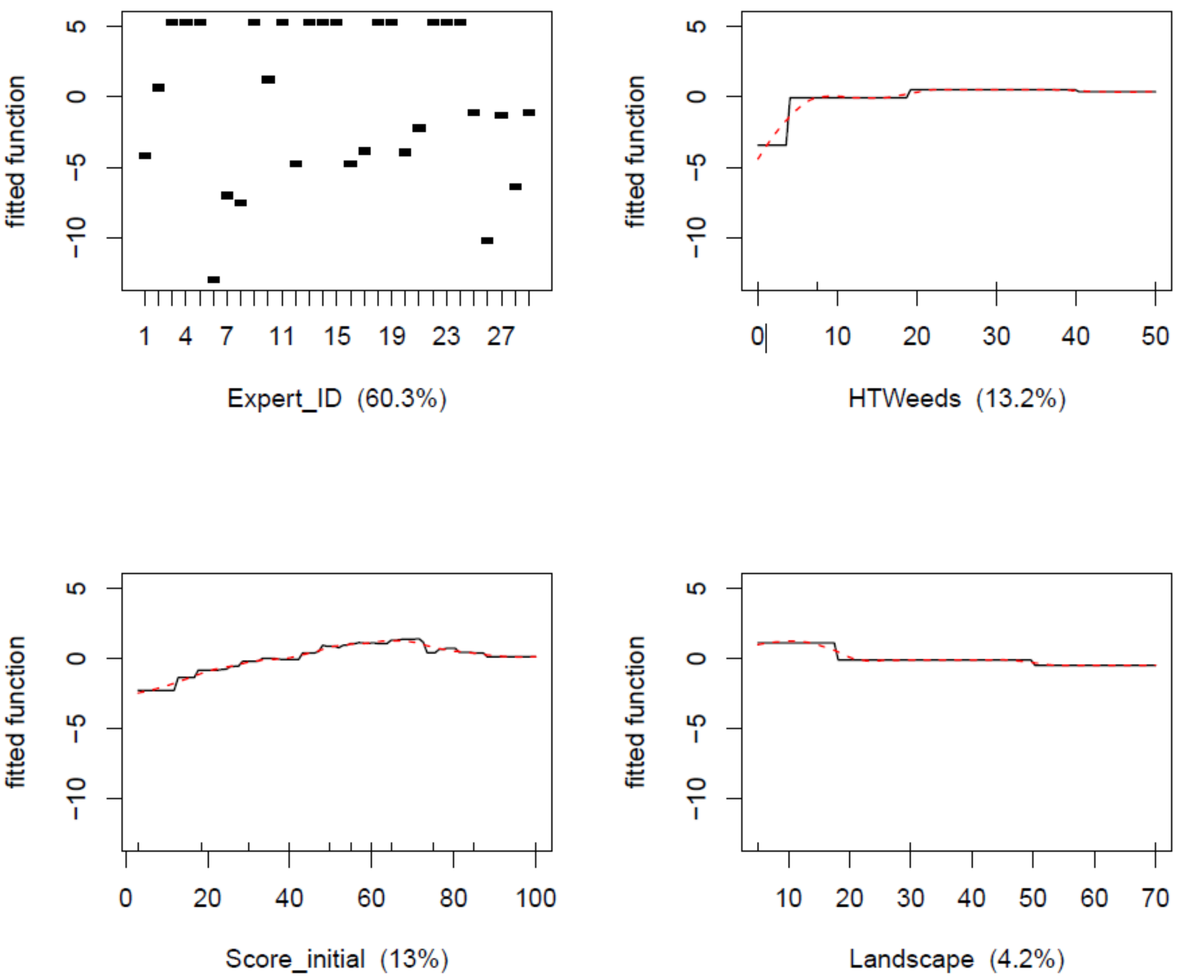

Fig S4.3 Fitted functions for expert aggregate averted loss (y) and plot attribute values (x), for each of the 4 variables with highest normalised importance (expert identity, invasive alien plant cover, initial aggregate condition score and landscape vegetation cover) in Boosted Regression Tree models.

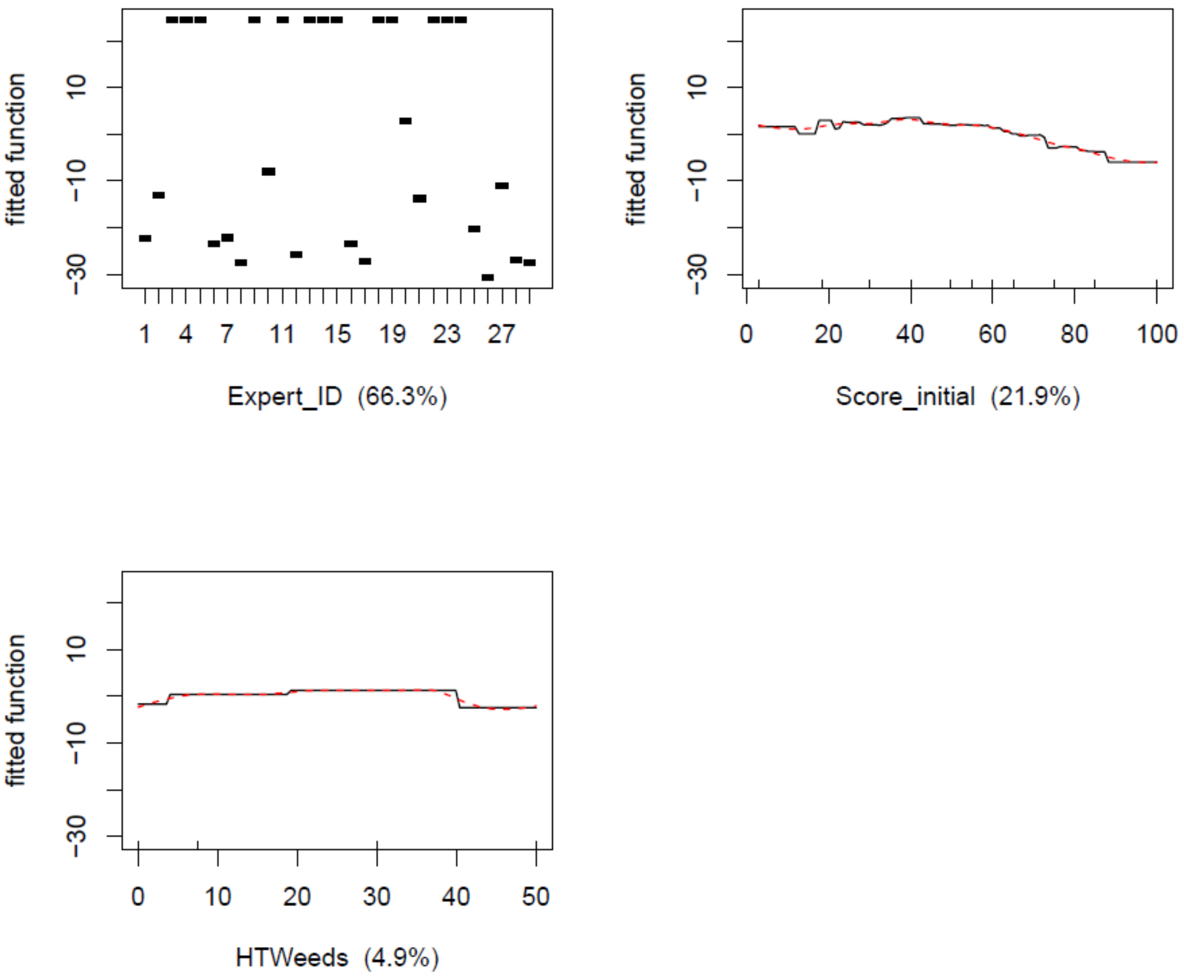

Fig S4.4 Fitted functions for expert aggregate total benefit (y) and plot attribute values (x), for each of the 3 variables with highest normalised importance (expert identity, initial aggregate condition score and invasive alien plant cover) in Boosted Regression Tree models.
