# Supplementary material for: Expert predictions of changes in vegetation condition reveal perceived risks in biodiversity offsetting
Source: PLoS One. 2019 May 8;14(5):e0216703. doi: 10.1371/journal.pone.0216703 (PMC6505952; doi:10.1371/journal.pone.0216703)
Supplement: S5 File — (PDF) [file pone.0216703.s005.pdf]

S5 Boxplots of individual expert estimates of aggregate management gain

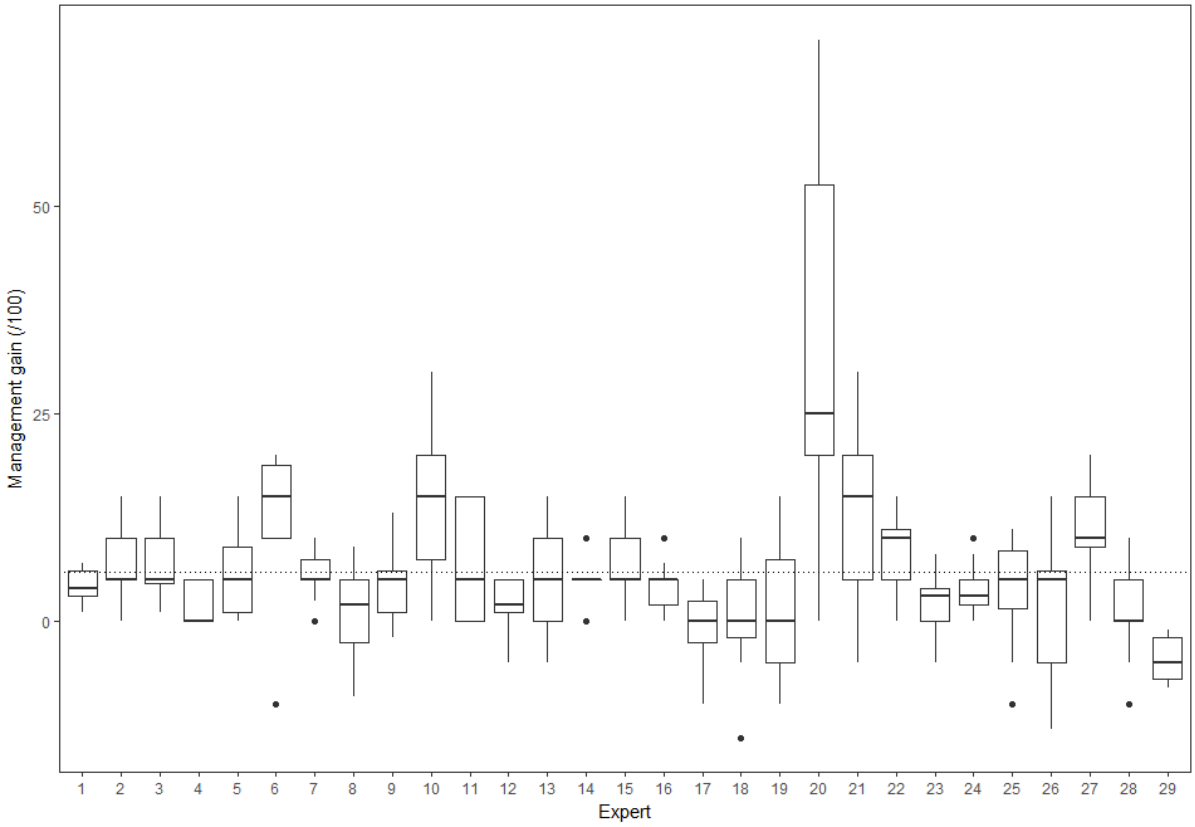

Fig S5 Boxplots for individual expert estimates of management gain (MG). For each expert data are derived from estimates of MG at 15 sites. The dotted line represents the overall mean MG across all experts and sites. Expert identity had the largest normalised importance in the BRT tree models of MG.
