# Supplementary material for: Expert predictions of changes in vegetation condition reveal perceived risks in biodiversity offsetting
Source: PLoS One. 2019 May 8;14(5):e0216703. doi: 10.1371/journal.pone.0216703 (PMC6505952; doi:10.1371/journal.pone.0216703)
Supplement: S6 File — (PDF) [file pone.0216703.s006.pdf]

S6 Boxplots of individual expert probability distributions of MG, AL and TB

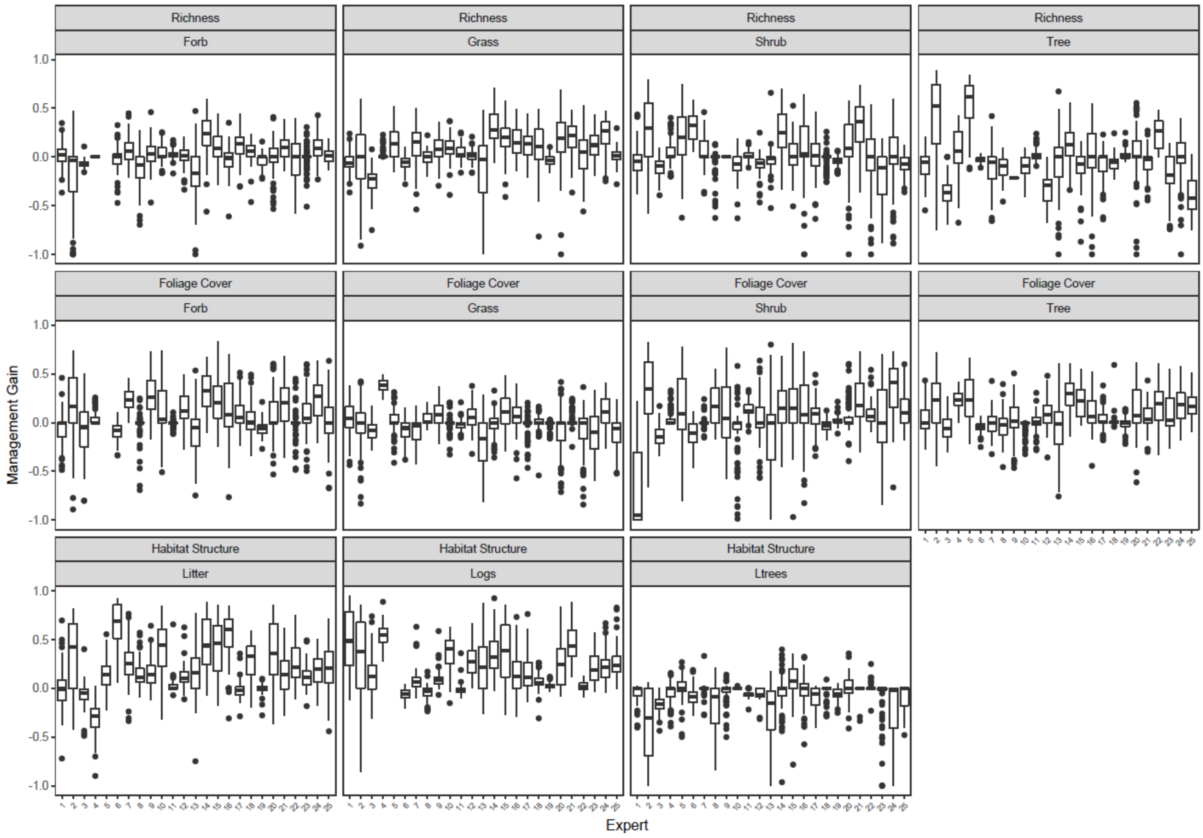

Fig S6.1 Boxplots of individual expert estimates of Management Gain for 11 attributes within a Western Slope Grassy Woodland. Values greater than 0 indicate that the expert expects that attribute values will increase following adoption of an offset. Estimates are obtained from randomly drawn samples of each expert's subjective probability distributions.

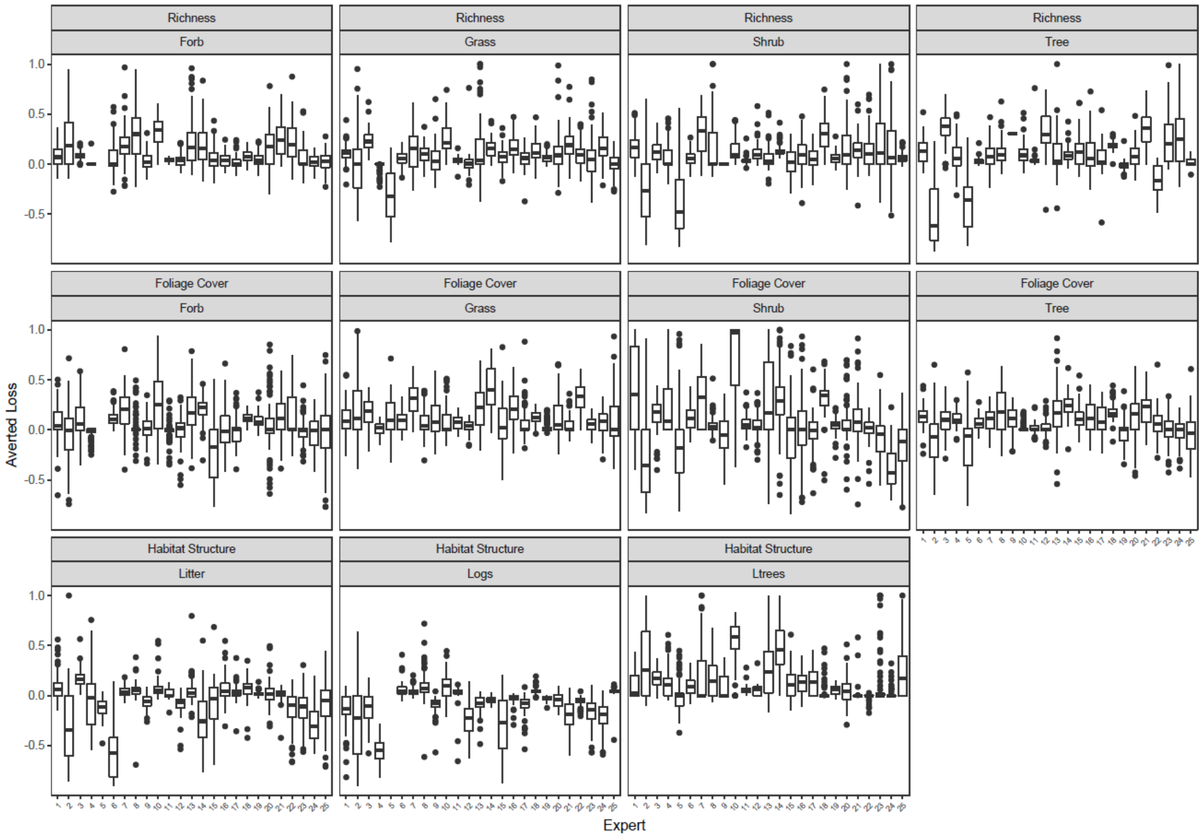

Fig S6.2 Boxplots of each experts estimates of Averted Loss for 11 attributes within a Western Slope Grassy Woodland. Values greater than 0 indicate that the expert expects that attribute values will decline in the absence of an offset. Estimates are obtained from randomly drawn samples of each expert's subjective probability distributions.

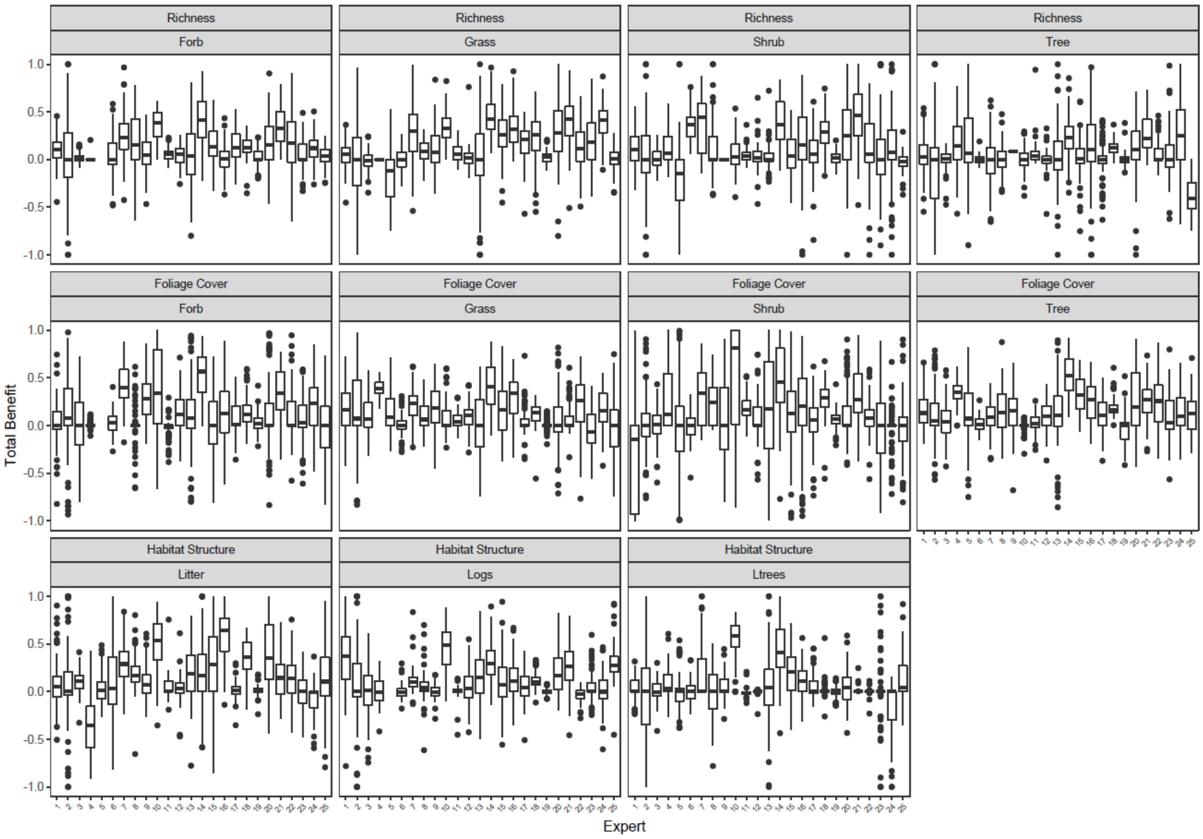

Fig S6.3 Boxplots of each experts estimates of Total Benefit for 11 attributes within a Western Slope Grassy Woodland. Total Benefit is the sum of Management Gain and Averted Loss and are the summed benefit experts expect to result from adoption of a biodiversity offset. Estimates are obtained from randomly dr
