# Supplementary material for: Expert predictions of changes in vegetation condition reveal perceived risks in biodiversity offsetting
Source: PLoS One. 2019 May 8;14(5):e0216703. doi: 10.1371/journal.pone.0216703 (PMC6505952; doi:10.1371/journal.pone.0216703)
Supplement: S7 File — (PDF) [file pone.0216703.s007.pdf]

S7 Individual expert mean estimates of Averted Loss and Management Gain. Are individual experts consistently optimistic or pessimistic?

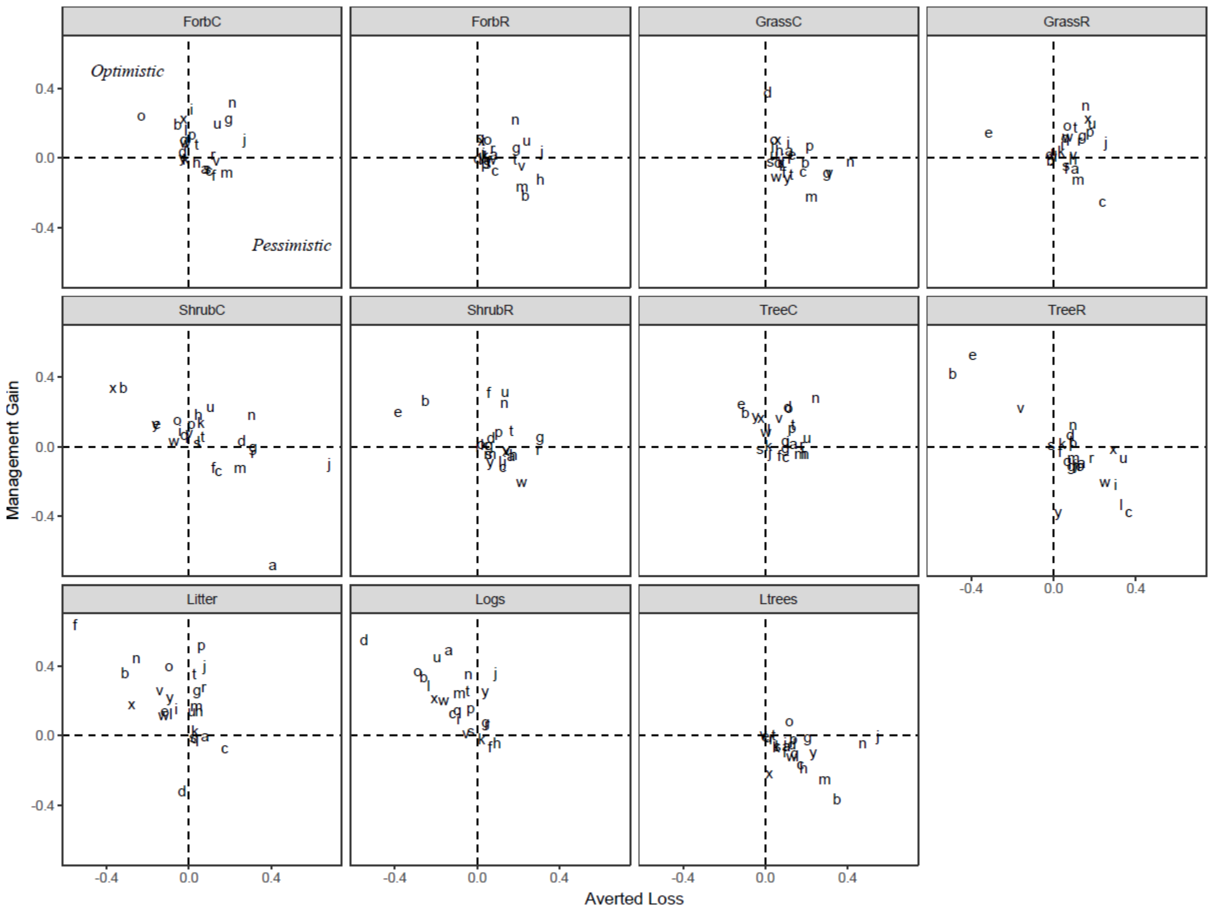

Fig S7.1 Relationship between individual expert average estimates of averted loss and management gain for each of 11 habitat attributes, with each expert represented by an alphabetic label. Pessimism is indicated by estimates of MG <0 and AL > 0, suggesting an expectation that values will decline in the presence or absence of a biodiversity offset. Optimism is indicated by estimates of MG >0 and AL <0. Most experts are not similarly optimistic or pessimistic about trends across all vegetation attributes although b, n and e tend to be more optimistic but not in all cases and c & m tend to be pessimistic.

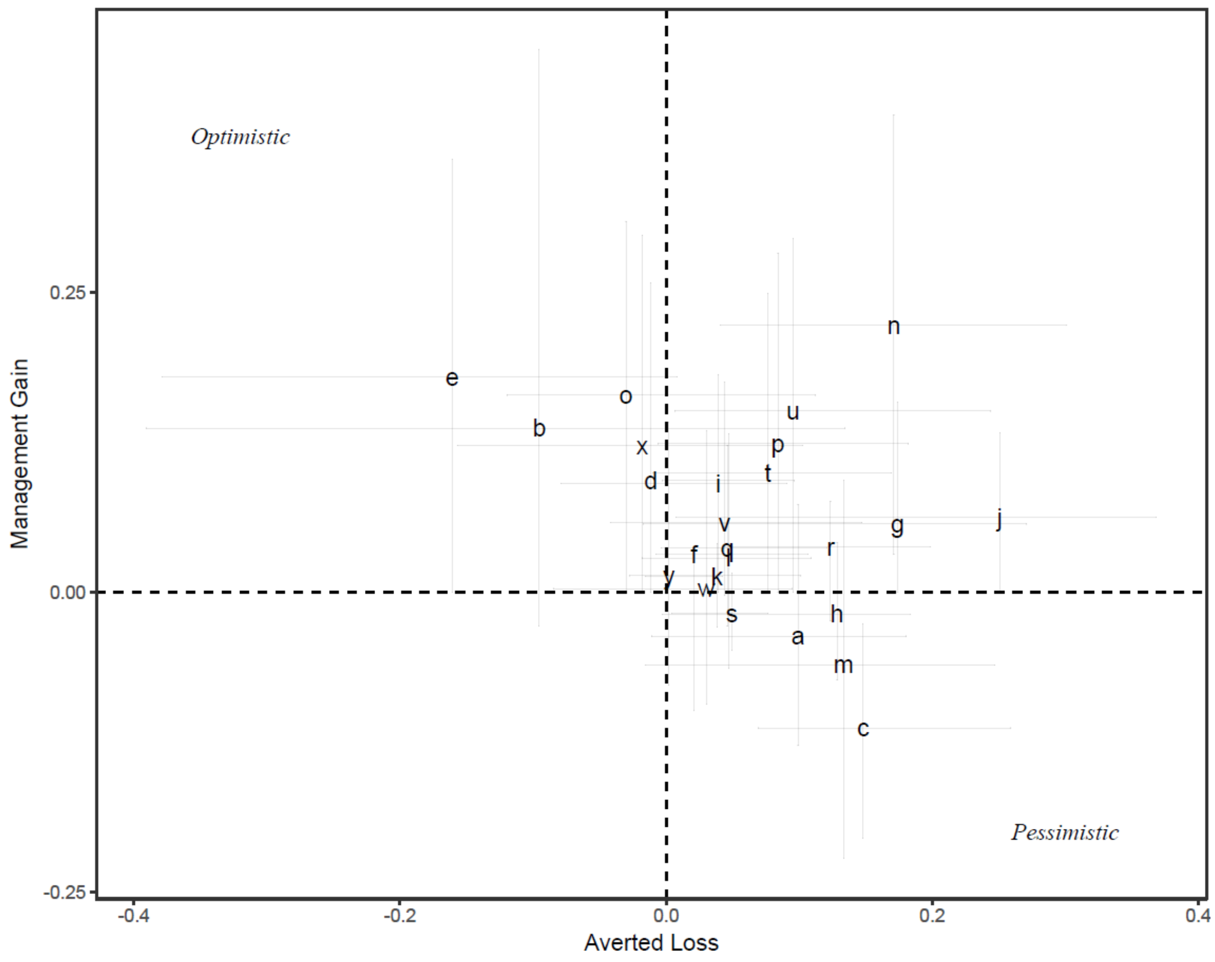

Fig S7.2 Expert estimates of averted loss and management gain, averaged over 11 vegetation attributes, with associated 25th and 75th percentiles. Values that fall in the top left corner indicate those experts that are generally optimistic about future trends, expecting on average vegetation will improve towards benchmarks regardless of management scenario. Those in the bottom right corner are generally pessimistic, believing that on average vegetation will tend to decline, relative the reference, under both BAU and offset management. Most experts on average are pessimistic about the future with BAU but neutral or positive about the future with an offset (top right quarter).
