# Supplementary material for: Expert predictions of changes in vegetation condition reveal perceived risks in biodiversity offsetting
Source: PLoS One. 2019 May 8;14(5):e0216703. doi: 10.1371/journal.pone.0216703 (PMC6505952; doi:10.1371/journal.pone.0216703)
Supplement: S8 File — (PDF) [file pone.0216703.s008.pdf]

S8 Boosted regression tree for predicting vegetation condition to new sites

An ensemble Boosted Regression Tree (BRT) model, based on 29 independent expert evaluations of 15 synthetic sites, were used to predict the aggregate initial vegetation condition of three WSGW scenarios. The 15 synthetic sites were drawn from a total pool of 64 sites. A BRT model was fit, including 13 individual vegetation attributes, total alien plant cover, invasive alien plant cover and landscape native vegetation cover. The model excluded individual expert. The model was fit with a tree complexity of 5, a learning rate of 0.005 and a bag fraction of 0.75. The predict function in gbm was used to develop predictions of vegetation condition for the three WSGW sites (see Supporting Information S4.B). These three sites were based on available plot data and were selected by the authors as potential examples of poor, moderate and good vegetation conditions. This *a priori* categorisation was confirmed through the predicted aggregate vegetation condition provided by the expert BRT ensemble model.
