# Supplementary material for: Expert predictions of changes in vegetation condition reveal perceived risks in biodiversity offsetting
Source: PLoS One. 2019 May 8;14(5):e0216703. doi: 10.1371/journal.pone.0216703 (PMC6505952; doi:10.1371/journal.pone.0216703)
Supplement: S9 File — (PDF) [file pone.0216703.s009.pdf]

## S9 Predicted change in individual attribute values with offset on original measurement scale

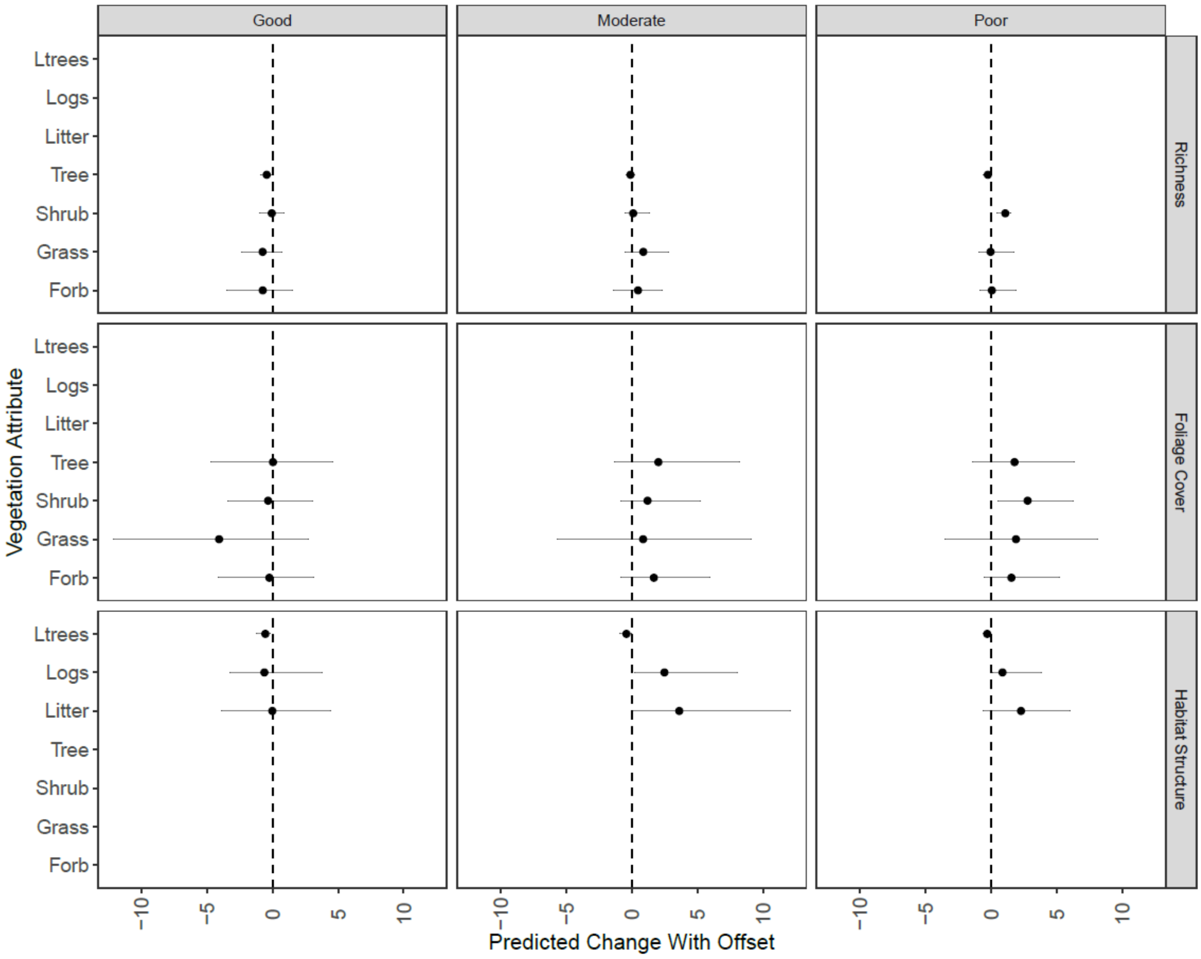


*Fig S9.1 Predicted change in the value of 11 attributes with offset, across all experts. Predictions are the median (point), 25th and 75th percentiles (error bars) on the original measurement scale (richness = number of species, foliage cover = % cover, Large Trees = individuals/0.1ha, Logs = lineal length in metres/0.1ha, litter= % cover) and are derived from 2100 randomly drawn samples. Results are for three Western Slopes Grassy Woodlands in poor, moderate and good condition (see Supporting Information S3.C & Supporting Information S7).*
